# Vascular and inflammatory biomarkers of cardiovascular events in non-steroidal anti-inflammatory drug users

**DOI:** 10.1093/ehjopen/oeae088

**Published:** 2024-11-02

**Authors:** Ricky Vaja, Plinio Ferreira, Laura Portas, Blerina Ahmetaj-Shala, Neringa Cypaite, Hime Gashaw, Jennifer Quint, Ramzi Khamis, Adam Hartley, Thomas M MacDonald, Isla S Mackenzie, Nicholas S Kirkby, Jane A Mitchell

**Affiliations:** The National Heart and Lung Institute, Imperial College London SW7 2AZ, UK; The Royal Brompton Hospital, London SW3 6NP, UK; The National Heart and Lung Institute, Imperial College London SW7 2AZ, UK; The National Heart and Lung Institute, Imperial College London SW7 2AZ, UK; The National Heart and Lung Institute, Imperial College London SW7 2AZ, UK; The National Heart and Lung Institute, Imperial College London SW7 2AZ, UK; The National Heart and Lung Institute, Imperial College London SW7 2AZ, UK; The National Heart and Lung Institute, Imperial College London SW7 2AZ, UK; The National Heart and Lung Institute, Imperial College London SW7 2AZ, UK; The National Heart and Lung Institute, Imperial College London SW7 2AZ, UK; Ninewells Hospital and Medical School, University of Dundee, Dundee DD2 1SG, UK; Ninewells Hospital and Medical School, University of Dundee, Dundee DD2 1SG, UK; The National Heart and Lung Institute, Imperial College London SW7 2AZ, UK; The National Heart and Lung Institute, Imperial College London SW7 2AZ, UK

**Keywords:** Non-steroidal anti-inflammatory, NSAID, Biomarkers, Cardiovascular

## Abstract

**Aims:**

The Standard care vs. Celecoxib Outcome Trial (SCOT) found similar risk of cardiovascular events with traditional non-steroidal anti-inflammatory drugs (NSAIDs) and the cyclooxygenase-2-selective drug celecoxib. While pre-clinical work has suggested roles for vascular and renal dysfunction in NSAID cardiovascular toxicity, our understanding of these mechanisms remains incomplete. A *post hoc* analysis of the SCOT cohort was performed to identify clinical risk factors and circulating biomarkers of cardiovascular events in NSAID users.

**Methods and results:**

Within SCOT (7295 NSAID users with osteoarthritis or rheumatoid arthritis), clinical risk factors associated with cardiovascular events were identified using least absolute shrinkage and selection operator regression. A nested case–control study of serum biomarkers including targeted proteomics was performed in individuals who experienced a cardiovascular event within 1 year (*n* = 49), matched 2:1 with controls who did not (*n* = 97). Risk factors significantly associated with cardiovascular events included increasing age, male sex, smoking, total cholesterol:HDL ratio ≥5, and aspirin use. Statin use was cardioprotective [odds ratio (OR) 0.68; 95% confidence interval (CI) 0.46–0.98]. There was significantly higher immunoglobulin (Ig)G anti-malondialdehyde-modified LDL (MDA-LDL), asymmetric dimethylarginine (ADMA), and lower arginine/ADMA. Targeted proteomic analysis identified serum growth differentiation factor 15 (GDF-15) as a candidate biomarker [area under the curve of 0.715 (95% CI 0.63–0.81)].

**Conclusion:**

Growth differentiation factor 15 has been identified as a candidate biomarker and should be explored for its mechanistic contribution to NSAID cardiovascular toxicity, particularly given the remarkable providence that GDF-15 was originally described as *NSAID-activated gene-1*.

## Graphical Abstract

**Graphical Abstract:**
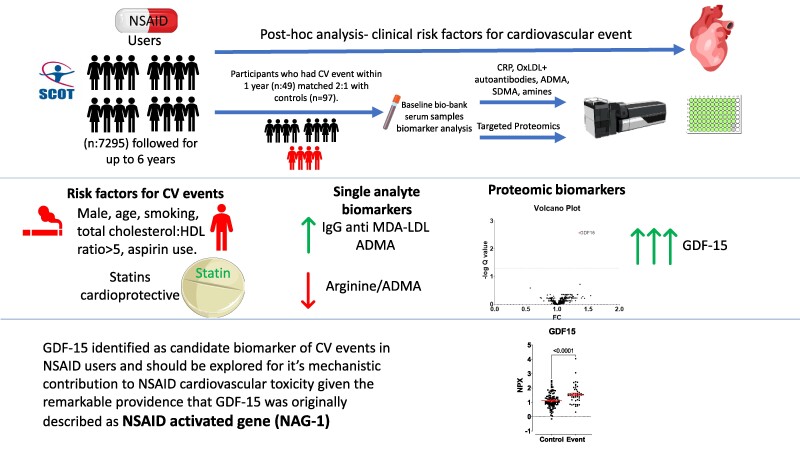

## Introduction

Cyclooxygenase (COX) is the first enzyme in the conversion of arachidonic acid to prostanoids, a group of lipid mediators with a diverse and broad range of homeostatic and inflammatory functions. Cyclooxygenase is expressed in two isoforms, COX-1 and COX-2. Cyclooxygenase-1 was the first isoform to be identified and is constitutively expressed in many cell types. Cyclooxygenase-2, on the other hand, is induced at the site of inflammation and is the therapeutic target for the non-steroidal anti-inflammatory drugs (NSAIDs).^1^

Non-steroidal anti-inflammatory drugs such as ibuprofen and diclofenac, sometimes referred to as ‘traditional or non-selective NSAIDs’, were formulated before the identification of COX-2 and as such inhibit both isoforms, a property that is associated with increased risk of gastrointestinal side effects.

Driven by the clinical need to spare the gut, after the discovery of COX-2, selective NSAIDs, such as celecoxib, were introduced in the early 2000s.^1^ However, shortly after their introduction, placebo-controlled trials indicated that use of COX-2-selective NSAIDs increased the risk of cardiovascular events. These findings together with subsequent pre-clinical data established that while ‘inducible’ COX-2 mediates pain and inflammation, a constitutively expressed form of COX-2 is a cardioprotective enzyme^2^ and that cardiovascular side effects of NSAIDs are attributed to the inevitable but unintentional inhibition of homeostatic COX-2.

Cyclooxygenase-1 in platelets drives the production of the pro-thrombotic prostanoid, thromboxane, which is the therapeutic target of low-dose aspirin. Since COX-2-selective drugs have minimal activity on COX-1 and indiscriminately inhibit both inducible and constitutive COX-2 at therapeutic doses, it was originally thought that cardiovascular side effects may be limited to this sub-class of NSAIDs.^2^ However, epidemiological data suggested that traditional NSAIDs carry similar risk of cardiovascular events as COX-2-selective drugs. To test this directly, prospective clinical trials were designed to compare the risk of cardiovascular adverse effects associated with the use of celecoxib in real-world settings where participants were deemed to be at either (i) low cardiovascular risk [from the Standard care vs. Celecoxib Outcome Trial (SCOT)^3^] or (ii) high cardiovascular risk (PRECISION.^4^) Results from both SCOT and PRECISION showed non-inferiority of celecoxib compared with traditional NSAIDs (SCOT) or ibuprofen and naproxen (PRECISION).

Pre-clinical work suggests that COX-2-derived prostacyclin protects the cardiovascular and renal system. However, the downstream mechanisms involved are incompletely understood, and there are no experimental or other biomarkers identified that associate with susceptibility to NSAID cardiovascular risk. Here, we have used data and serum samples from SCOT in a nested case–controlled study to establish biomarkers associated with cardiovascular events in this cohort of patients.

In our analysis, we have included components of clinical risk factors, circulating markers of endothelial dysfunction, and a cardiometabolic-targeted proteomic array.

## Methods

### Study population

Standard care vs. Celecoxib Outcome Trial was a prospective, multi-centre, randomized, open-label clinical trial comparing the gastrointestinal and cardiovascular safety of a prescribed traditional NSAID vs. switching to prescribed celecoxib in patients with low cardiovascular risk with rheumatoid arthritis or osteoarthritis. A detailed protocol^5^ and primary results have already been published.^3^

Both men and women were eligible for inclusion if they were over 60 years of age, chronic NSAID users (≥ 90 days or at least three filled prescriptions in the last year), and free from established cardiovascular disease. Exclusion criteria included ischaemic heart disease, myocardial infarction, angina or acute coronary syndrome, cerebrovascular disease including cerebrovascular accident or transient ischaemic attack, peripheral vascular disease or moderate/severe heart failure, patients who had taken selective COX-2 inhibitors within 90 days of screening, life threatening comorbidity, clinically significant renal or hepatic disease, patients who were unlikely to comply with medications, allergy to NSAIDs, hypersensitivity to sulfonamide antibiotics, active peptic ulceration or gastrointestinal bleeding, patients scheduled to have arthritis surgery, and those participating in another clinical trial.

The trial was conducted in the UK, the Netherlands, and Denmark between January 2008 and March 2013 across 9 trial centres and 706 primary care practices. Eligible patients were identified in a primary care setting and were randomized to either switch to celecoxib or remain on their usual traditional NSAID prescription. The medications were prescribed at approved doses and adjusted according to clinical indication. Serum samples were collected at the screening visit and stored in a biobank.^5^

### Clinical risk predictors

A detailed description of the methodology and statistical analysis used to assess clinical predictors of cardiovascular events is provided in Supplementary material online, *Section S1*. In brief, cases from the SCOT population^3^ were defined as patients who had reached the primary endpoint, which was a composite of hospitalization for non-fatal myocardial infarction or biomarker of positive acute coronary syndrome, non-fatal stroke, or cardiovascular death. Controls were identified as patients who did not reach the primary endpoint. Candidate predictors of cardiovascular events for use in the logistic model were selected using the least absolute shrinkage and selection operator (LASSO) method.^6^ This study is reported according to the Transparent Reporting of a Multivariable Prediction Model for Individual Prognosis or Diagnosis statement for developing and validating multivariable prediction models.^7^

### Sex as a biological variable

Both male and female sexes were considered as biological variables and were reported.

### Biomarker discovery; case–control study

Within the full SCOT cohort, a case–control study was designed to identify biomarkers that were associated with cardiovascular events in the NSAID users and elucidate novel mechanisms by which COX-2 protects the cardiovascular system.

In SCOT, there were ∼7000 participants who were followed up for up to 6 years, with a median follow-up of 3.6 years. As biological changes in serum markers are likely more apparently closer to the time of a cardiovascular event, this study considered only participants who had events within the first year of recruitment.

### Study design

Cases were identified as participants who had reached the primary endpoint within 1 year. To minimize the risk of selection bias, the pool of patients for control sampling had at least 1 year follow-up. Furthermore, participants who went on to have an event after 1 year were still included in the pool of controls. Participants who had rheumatoid arthritis were not included as the autoimmune disease pathology in these individuals may confound interpretation of biomarker findings. Furthermore, data relating to the use of disease-modifying anti-rheumatic drugs (DMARDs) were not available.

There were 50 eligible cases in the cohort, with serum samples available for 49 of these. These were matched 2:1 to controls using the SPSS extension package Fuzzy matching. Variables were matched exactly using sex, country, and baseline NSAID. Age had a deviation of ±5 years whereby the software first prioritizes exact matches. When this was not possible, the closest remaining match was identified (without replacement to ensure each participant can only act as one control). If there was a tie between matches, the software randomly allocated one. One serum sample in the control group was not available, and so in total, there were 49 cases and 97 controls. Baseline characteristics for the case–control study are provided in Supplementary material online, *Table S1*.

### Biomarker analysis

A detailed description of the methodology for each of the platforms used for biomarker analysis is provided in Supplementary material online, *Section S2*. In summary, malondialdehyde-modified LDL (MDA-LDL), apolipoprotein B (ApoB), IgM anti-MDA-LDL, IgG anti-MDA-LDL, and C-reactive protein (CRP) were measured by enzyme-linked immunosorbent assay; methylarginines and amines were measured using ultra-high performance liquid chromatography tandem mass spectrometry; and targeted proteomics was performed using the Olink Explore 384 Cardiometabolic panel.

The 146 samples were spread across two 96-well plates. In order to account for any potential batch effect, event samples were evenly spread across the two plates along with the matched controls, with samples randomly aliquoted on each plate. The operator conducting the assay was blinded to the groups. To keep sample condition as controlled as possible, an additional freeze thaw step was introduced where required.

### Statistical analysis

Data in figures are presented as individual data points and/or mean ± standard error of the mean. Normality was assessed using the Shapiro–Wilk test and Kolmogorov–Smirnov tests. Visual distribution of the data was also confirmed using histograms and quantile-quantile plots. Normally distributed data were analysed using unpaired *t*-test, and non-normally distributed data were analysed using Mann–Whitney U-test. Graphs were generated using GraphPad Prism. Principal component analysis was performed using SIMCA 13.0.03 (Sartorius Stedim Biotech, Aubagne, France).

Regression and receiver operator characteristics (ROC) analyses were performed using IBM SPSS. Data are presented as odds ratios (ORs) alongside 95% confidence intervals (CIs). Least absolute shrinkage and selection operator was performed using R version: 4.0.3, R package, ‘glmnet’.

A two-tailed significance of *P* < 0.05 was deemed to be statistically significant. For proteomic analysis, *P*-values were obtained as above followed by the application of a false discovery correction using the Benjamini, Krieger, and Yekutieli method with *q* values provided.

### Study approval

This study was approved by the Imperial College Research Ethics Committee on 06 January 2020 (Ref: 19IC5645). The SCOT study was approved by the UK Multi-Centre Research Ethics Committee (reference number: 2006-005735-24) as well as by relevant authorities in Denmark and the Netherlands. Participants provided written informed consent. The research was conducted in accordance with the principles in the Declaration of Helsinki and in accordance with local statutory requirements.

## Results

### Clinical risk predictors

In the primary SCOT analysis,^3^ there were no significant subgroup interactions of pre-defined covariates with cardiovascular events between the standard care or celecoxib group and no difference in the primary outcome when stratifying for the different types of baseline NSAID (ibuprofen, diclofenac, and other NSAIDs).

Here, we have conducted a new analysis to explore covariates between all NSAID users who had a cardiovascular event vs. those who did not.

Within the SCOT dataset, 7297 NSAID users were identified of which 249 participants (3.49%) in the intention to treat group reached the primary endpoint. The median follow-up was 3.6 years (total of 22 600 years, maximum 6.3 years). Baseline characteristics of individuals in the event and non-event groups are provided in Supplementary material online, *Table S2*. Next, LASSO was used, which did not identify any further variables for exclusion. The 11 variables included in the final model were age, sex, smoking status, systolic blood pressure (BP) at screening, rheumatoid arthritis, diabetes, waist:hip ratio >1, aspirin use, statin use, total cholesterol:HDL ratio >5, and serum uric acid. In 771 cases (10.6%), there was at least one variable missing. In the final regression analysis, the total number of cases included 226 cases (3.6%) and 6300 controls. A summary is presented in *Table 1*.

**Table 1:** Factors associated with a cardiovascular event using binary logistic regression

Variable	*β* coefficient	SE	OR	95% CI for OR		*P*-value
				Lower	Upper	
Sex						
Female	Ref					
Male	0.57	0.156	1.77	1.303	2.404	0.0003
Smoking status						
Non-smoker	Ref					
Current smoker	0.905	0.189	2.47	1.706	3.579	<0.0001
Former smoker	0.253	0.167	1.288	0.928	1.787	0.131
Systolic blood pressure at screening						
<130 mmHg	Ref					
130–159 mmHg	0.044	0.168	1.045	0.752	1.453	0.792
≥160 mmHg	0.362	0.216	1.436	0.94	2.19	0.094
Arthritis						
Osteoarthritis	Ref					
Rheumatoid arthritis	0.478	0.252	1.613	0.985	2.642	0.058
Diabetes						
No	Ref					
Yes	0.438	0.228	1.55	0.99	2.425	0.055
Concomitant aspirin use						
No	Ref					
Yes	0.422	0.185	1.525	1.060	2.193	0.023
Total cholesterol:HDL ratio						
<5	Ref					
≥5	0.674	0.173	1.961	1.397	2.754	0.0001
Age						
60–69 years	Ref					
70–79 years	0.499	0.152	1.647	1.223	2.219	0.001
≥80 years	1.401	0.22	4.059	2.637	6.248	<0.0001
Statin use						
No	Ref					
Yes	−0.394	0.193	0.675	0.463	0.984	0.041
Uric acid						
<300 μmol/L	Ref					
300–400 μmol/L	−0.085	0.162	0.919	0.669	1.262	0.601
>400 μmol/L	0.078	0.192	1.082	0.742	1.576	0.683
Waist:hip ratio						
<1	Ref					
≥1	0.051	0.183	1.053	0.736	1.506	0.779
Constant	−4.439	0.216	0.012			<0.0001

The model includes all variables listed (male gender, smoking status, systolic blood pressure, arthritis, diabetes, waist:hip ratio, statin use, aspirin use, total cholesterol:HDL ratio, uric acid, and age). Each variable is adjusted for all other variables shown. The total number of participants used in the analysis was 6300 controls and 226 cases (3.6%).

SE, standard error; OR, odds ratio; CI, confidence interval; Ref, reference.

Clinical risk factors significantly associated with cardiovascular events included increasing age, male sex, current smoking, total cholesterol:HDL ratio ≥5, and concomitant aspirin use. The odds of a cardiovascular event were lower in statin users (OR 0.68; 95% CI 0.46–0.98). While not statistically significant, there was a trend for patients with diabetes, rheumatoid arthritis, and systolic BP ≥160 mmHg being more likely to have cardiovascular events.

While data from epidemiological studies along with prospective trials (e.g. SCOT and PRECISION) suggests that the risk of cardiovascular events is shared across all NSAID medications, we have explored differences in risk factors according to individual NSAIDs. Data were stratified according to the NSAID allocated during the trial and was presented separately for each NSAID in Supplementary material online, *Table S3*. Given this is a subgroup analysis, with a relatively low number of events within each subgroup, extreme caution should be undertaken when interpreting the results. However, the authors want to highlight an interesting observation; the odds of a cardiovascular event were significantly higher in ibuprofen users who had a systolic BP ≥160 mmHg (OR 4.4; 95% CI 1.6–12.1, *P* = 0.004).

### Circulating biomarkers

We took two approaches in our investigation of circulating biomarkers. First, we measured levels of rationally selected analytes with established associations with cardiovascular risk including CRP,^8^ oxidized LDL (OxLDL) (MDA-LDL) and related autoantibodies,^9^ the methylarginines [asymmetric dimethylarginine (ADMA)^10^ and symmetric dimethylarginine (SDMA^11^)], and arginine along with other amino acids. Second, to identify novel biomarkers, we measured 369 cardiometabolic proteins using Olink targeted proteomic platform. In each case, levels were quantified in serum samples from 49 participants with events and 97 controls.

### C-reactive protein

**Figure 1:**
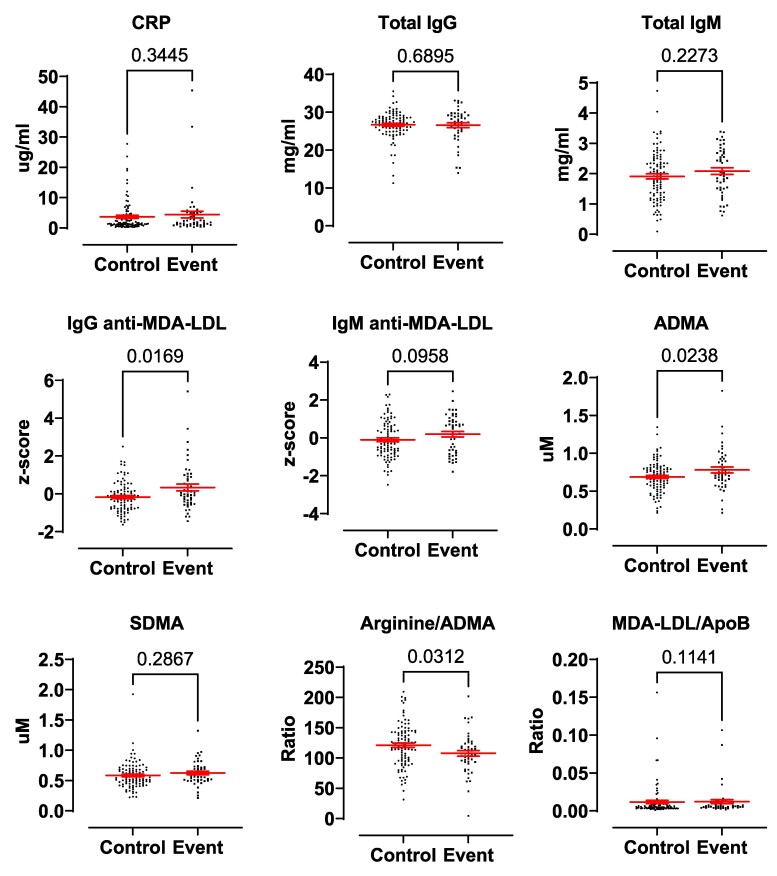
Scatter plots of circulating biomarkers with established associations with cardiovascular risk. Data are presented as individual points. The error bars represent standard error of the mean. Mann–Whitney U-test was used to analyse C-reactive protein, total IgG, IgG anti-malondialdehyde-modified LDL, and malondialdehyde-modified LDL/apolipoprotein B. All other analytes were analysed using an unpaired *t*-test. C-reactive protein, IgG anti-malondialdehyde-modified LDL, IgM anti-malondialdehyde-modified LDL, and malondialdehyde-modified LDL/apolipoprotein B: *n* = 144 participants (95 controls and 49 events); total IgG, total IgM, asymmetric dimethylarginine, symmetric dimethylarginine, and arginine/asymmetric dimethylarginine: *n* = 146 participants (97 controls and 49 events).

Levels of CRP, which is a well-established marker of inflammation and cardiovascular disease, did not differ between the event and non-event groups (*Figure 1*).

### Malondialdehyde-modified LDL and associated autoantibodies

Levels of OxLDL, measured as MDA-LDL, did not differ between groups. Levels of IgG anti-MDA-LDL (*P* = 0.017) were significantly higher in the event group, and while not statistically significant, immunoglobulin (Ig)M anti-MDA-LDL (*P* = 0.096) showed a tendency towards elevation in the event group. This was not explained by a general level of elevated antibodies since total IgG or IgM was not different in serum from the two groups (*Figure 1*). There was no difference in levels of MDA-LDL after adjusting for ApoB between controls and events (*P* = 0.114).

### Methylarginines

Levels of ADMA, a competitive inhibitor of endothelial nitric oxide synthase (NOS) and marker of renal dysfunction, were increased in serum from patients who went on to have events compared with controls. By contrast, levels of the inactive isomer, SDMA, were not altered. Levels of arginine, the substrate for NOS, in serum from the event group did not differ from controls. In agreement, arginine/ADMA ratio was correspondingly lower in the event group (*Figure 1*). There were no differences in levels of other amines measured (see Supplementary material online, *Table S4* for full list).

### Targeted proteomics

**Figure 2:**
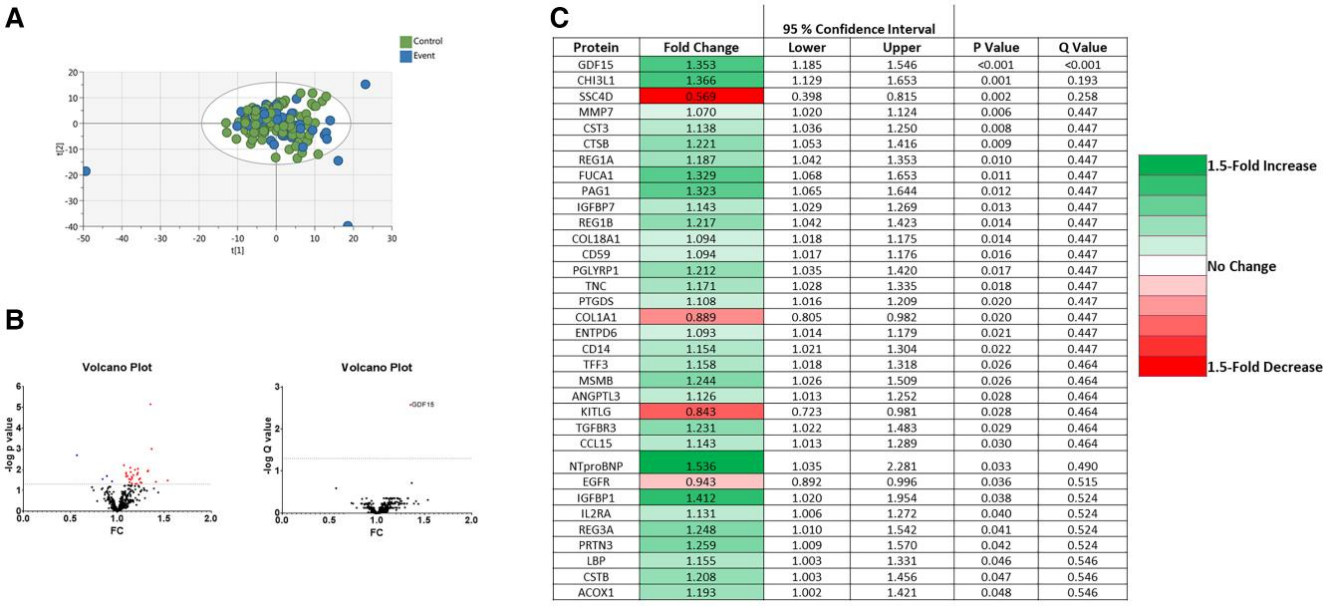
Targeted cardiometabolic proteomic analysis. (*A*) Principal component analysis with samples labelled according to group (control: green, event: blue). (*B*) Volcano plots showing proteins that are significantly different between events and controls before and after false discovery correction using the Benjamini, Krieger, and Yekutieli method. At a *P*-value cut-off of <0.05 (dotted line), 34 proteins were significantly different including 4 down- (blue) and 30 up-regulated (red). After applying FDR at a Q value cut-off of <0.05 (dotted line), only growth differentiation factor 15 remained significant. An unpaired *t*-test was used, *n* = 146 participants (49 events and 97 controls). (*C*) Heat map of 34 proteins that were significantly altered. Proteins are presented in order of *P*-value. Dark green indicates a 1.5-fold increase, and dark red indicates a 1.5-fold decrease.

**Figure 3:**
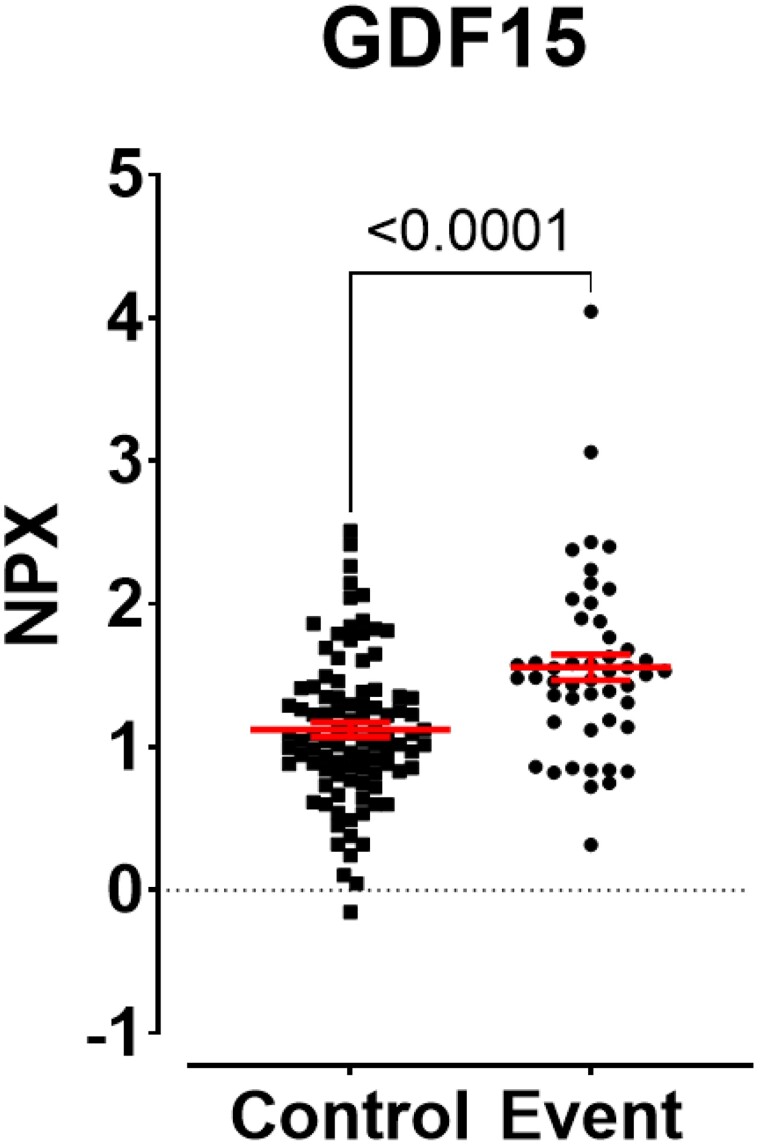
Scatter plot of growth differentiation factor 15. Data are presented as individual points. The error bars represent standard error of the mean. An unpaired *t*-test was used. *n* = 146 participants (97 controls and 49 events).

Targeted proteomic analysis of 369 cardiometabolic proteins was performed. An unsupervised approach using principal component analysis (PCA-X) identified no separation between the groups (*Figure 2A*). Using a supervised approach and a discovery *P*-value cut-off of <0.05, 34 proteins were significantly different between events and controls including 30 up-regulated and 4 down-regulated proteins (*Figure 2B*). These included proteins involved in atherosclerosis/inflammation (e.g. GDF-15, lipopolysaccharide binding protein, and CD14), extracellular matrix [matrix metalloproteinase-7 (MMP7)], heart failure [N-terminal pro-B-type natriuretic peptide (NT-proBNP)], and renal insufficiency cystatin C (CST3; *Figure 2C*). While these findings may provide some insight for future mechanistic investigations, following application of a false discovery rate correction, only one protein, growth differentiation factor 15, remained statistically significant (*q* < 0.01; *Figure 3*). Further details for all proteins analysed can be found in Supplementary material online, *Table S5*.

### Biomarkers of cardiovascular events in non-steroidal anti-inflammatory drug users

**Figure 4:**
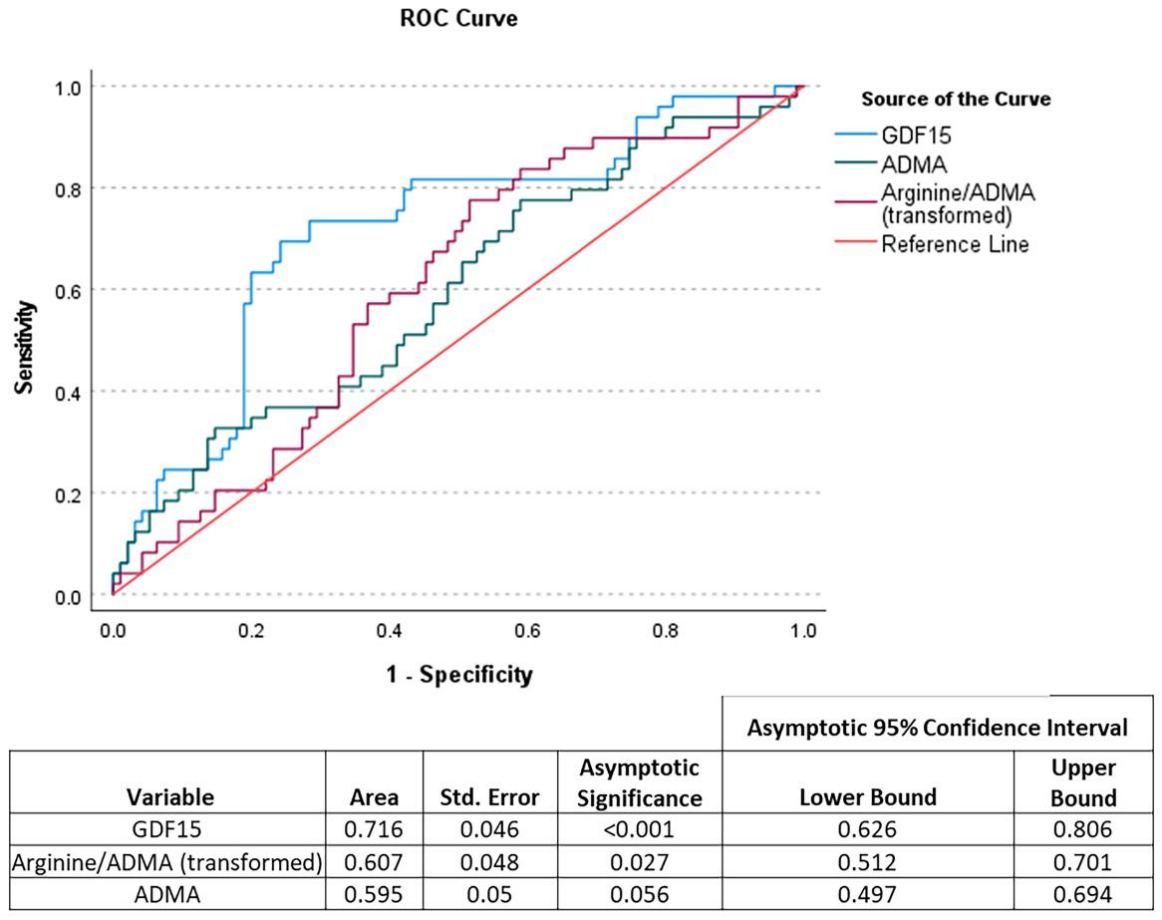
Receiver operating characteristic curves and area under the curve table. The proteins are presented in order of area under the curve. Arginine/asymmetric dimethylarginine was lower in the event group, and so data were transformed by multiplying values by −1 to display all variables on a single table. *n* = 146 participants (97 controls and 49 events).

Receiver operator characteristic analysis was performed on candidate biomarkers, which included all 34 proteins that were statistically significant (*Table 2*) and ADMA and arginine/ADMA ratio, Scavenger Receptor Cysteine Rich Family Member With 4 Domains (SSC4D), Collagen, type I, alpha 1 (COL1A1), epidermal growth factor receptor (EGFR), stem cell factor (KITLG), and arginine/ADMA were lower in the event group, and so data were transformed by multiplying values by −1 to display all variables on a single graph/table. Growth differentiation factor 15 was the best performing discriminator with an area under the curve (AUC) of 0.715 (95% CI 0.63–0.81). Receiver operator characteristic curves for GDF-15, ADMA, and Arginine/ADMA are provided in *Figure 4*.

**Table 2:** Area under the curve table

				95% confidence interval	
Variable	Area	SE	Asymptotic significance	Lower bound	Upper bound
GDF15	0.715	0.046	<0.001	0.625	0.805
IGFBP7	0.66	0.047	0.001	0.569	0.751
CHI3L1	0.658	0.049	0.001	0.563	0.753
SSC4D (transformed)	0.648	0.047	0.002	0.556	0.74
FUCA1	0.641	0.048	0.004	0.546	0.735
MMP7	0.638	0.048	0.004	0.544	0.732
CTSB	0.637	0.048	0.005	0.542	0.732
TNC	0.629	0.048	0.007	0.535	0.722
REG1A	0.626	0.048	0.009	0.531	0.72
CST3	0.62	0.048	0.012	0.526	0.713
TFF3	0.62	0.049	0.016	0.523	0.716
CD14	0.618	0.049	0.016	0.522	0.713
ENTPD6	0.616	0.049	0.019	0.519	0.712
COL18A1	0.615	0.047	0.014	0.524	0.707
PGLYRP1	0.61	0.049	0.024	0.514	0.706
REG1B	0.607	0.049	0.03	0.51	0.704
TGFBR3	0.607	0.049	0.03	0.51	0.703
MSMB	0.605	0.05	0.035	0.508	0.703
ANGPTL3	0.605	0.049	0.033	0.509	0.702
IGFBP1	0.604	0.052	0.047	0.501	0.706
PAG1	0.601	0.051	0.048	0.501	0.701
CD59	0.6	0.049	0.039	0.505	0.696
PTGDS	0.6	0.048	0.038	0.505	0.695
REG3A	0.598	0.05	0.05	0.5	0.697
NT-proBNP	0.596	0.05	0.053	0.499	0.693
CSTB	0.595	0.049	0.052	0.499	0.69
LBP	0.594	0.049	0.055	0.498	0.69
CCL15	0.592	0.052	0.075	0.491	0.693
IL2RA	0.589	0.053	0.094	0.485	0.694
COL1A1 (transformed)	0.587	0.05	0.079	0.49	0.684
EGFR (transformed)	0.587	0.051	0.09	0.486	0.688
KITLG (transformed)	0.584	0.051	0.096	0.485	0.684
PRTN3	0.583	0.05	0.099	0.485	0.681
ACOX1	0.554	0.052	0.302	0.452	0.656

The proteins are presented in order of area under the curve. SSC4D, COL1A1, EGFR, and KITLG were lower in the event group, and so data were transformed by multiplying values by −1 to display all variables on a single table. *n* = 146 participants (97 controls and 49 events).

SE, standard error.

### Adjusting for clinical differences did not alter key biomarker findings

The clinical risk factors in the overall SCOT cohort previously described in *Table 1* included age, male sex, current smoking, cholesterol, concomitant aspirin use, statin use (protective), diabetes, rheumatoid arthritis, and systolic BP. In the case–control study, samples were already matched by age, sex, country, and baseline NSAID. The baseline characteristics table suggested that while the overall matching between events and controls was sufficient, there were differences in the groups with regards to diabetes, smoking, and cholesterol. Differences in GDF-15, ADMA, arginine/ADMA ratio, and IgG anti-MDA-LDL persisted even after adjusting for diabetes, smoking, and cholesterol (*Table 3*).

**Table 3:** Circulating analytes associated with a cardiovascular event after adjustment for clinical variables using logistic regression

Variable	*β* coefficient	SE	OR	95% CI for OR		*P*-value
				Lower	Upper	
GDF15	1.42	0.42	4.13	1.83	9.3	<0.001
ADMA	2.1	0.84	7.75	1.49	40.5	0.015
Arginine/ADMA	−0.02	0.006	0.985	0.974	0.997	0.013

Variables presented in the table were adjusted for diabetes, smoking, and cholesterol. Data are presented as OR along with the 95% CI. *n* = 146 participants (97 controls and 49 events).

OR, odds ratio; CI, confidence interval; SE, standard error.

## Discussion

To our knowledge, this is the first study that has combined clinical data with biomarker analysis in NSAID users who later experienced a cardiovascular event. Of the serum analytes identified, GDF-15 had the strongest relationship with an AUC of 0.715, which remained highly significant after adjusting for clinical differences. The potential importance of GDF-15 in predicting cardiovascular events in this group clearly warrants future investigation. However, what makes GDF-15 either mechanistically logical or a remarkable coincidence is the fact that GDF-15 was first described as NSAID-activated gene-1 (NAG-1).

### Established clinical drivers of cardiovascular events in non-steroidal anti-inflammatory drug users

While it is unsurprising that well-established predictors of cardiovascular events in all populations such as smoking, diabetes, rheumatoid arthritis, cholesterol, sex, and age were also predictors of cardiovascular events among NSAID users, this study has identified several important findings that warrant further investigation.

### Aspirin use

Concomitant aspirin use was associated with an increased likelihood of cardiovascular events compared with non-users (OR 1.5; 95% CI 1.1–2.2). While a key inclusion criterion for SCOT was low cardiovascular risk, aspirin use was documented in some subjects, although data for the exact reason were not available. The deleterious effects of aspirin maybe be explained by bias due to indication of use; however, an opposite direction of effect was seen in statin users.

While there are no randomized controlled trials, a secondary analysis of the PRECISION study^12^ found that aspirin exposure among patients with rheumatoid arthritis with chronic NSAID use was associated with no significant increase in NSAID gastric or renal toxicity [hazard ratio (HR) 1.08; 95% CI 0.69 patients –1.69] or the risk of major adverse cardiovascular events (MACE) (HR 1.23; 95% CI 0.72–2.10]. Given the low quality of the available evidence, there remains a level of uncertainty surrounding aspirin use with NSAIDs and cardiovascular events, which should be addressed given the widespread use of these drugs.

### Statin use

Interestingly, the odds of a cardiovascular event were lower in statin users (OR 0.68; 95% CI 0.46–0.98). Clearly, statins are remarkably effective in preventing cardiovascular events in individuals with cardiovascular disease.^13^ However, while logical, it cannot be assumed that statins are protective in every clinical scenario, and to our knowledge, there are currently no studies that have explored the potential cardioprotective benefits of statins among chronic NSAID users. Participants in SCOT were over 50 years old and so would be included if the current public health drive for all individuals of this age or above were to be prescribed statins as a primary preventative strategy^13^. However, whether such an approach is adopted or not, our findings provide a rationale for the take up of statin prescriptions in those on NSAIDs, regardless of cardiovascular risk.

### Circulating biomarkers

We measured circulating levels of three key markers of endothelial function/cardiovascular risk including CRP, an established indicator of inflammation, the methylarginine ADMA, an endogenous inhibitor of endothelial NOS, and members of the OxLDL pathway, as well as performing a proteomic analysis of cardiometabolic mediators. It should be remembered that all subjects had osteoarthritis and were taking an NSAID at the time of serum collection. Thus, any ubiquitous baseline effect of disease and/or pharmacological block of prostanoids would be common across all participants. As such our study aimed to identify delineators of underlying pathways, which define specifically those individuals in this group who are at risk.

#### Methylarginines and amines

Increased levels of ADMA and/or reduction in the arginine:ADMA ratio is considered to indicate endothelial dysfunction and associates with renal dysfunction and increased risk of cardiovascular events. We have previously found that inhibition or genetic deletion of COX-2 increases ADMA levels in models where renal function is compromised.^14^ In this study, we found that ADMA levels were higher, and arginine:ADMA ratio was lower in event samples compared with controls. Together these findings suggest that reduced endothelial NOS capacity and thereby endothelial function may feature as a contributory factor to increased risk of cardiovascular events in patients taking NSAIDs.

#### Oxidized LDL

Oxidized LDL is a firmly established mediator of atherosclerosis and cardiovascular disease. It is immunogenic, generating detectable levels of specific IgG and IgM antibodies. We measured OxLDL as MDA-LDL and associated autoantibodies, and we found no differences in circulating levels of MDA-LDL or ApoB. However, we found higher levels of IgG anti-MDA-LDL (*P* = 0.017) and IgM anti-MDA-LDL (*P* = 0.096) in samples from patients with events compared with controls. The apparent increase in IgG anti-MDA-LDL was not accounted for by general atopy since there was no difference in total IgG (or IgM) between the groups. Anti-MDA-LDL antibodies have been shown to be both predictive and/or protective in cardiovascular disease, with differences between IgG and IgM serotypes being noted.^9^ However, increased levels of IgG anti-MDA-LDL have been associated with major adverse cardiovascular events.^15^

#### Targeted proteomics

While we identified 34 proteins altered in the serum of patients experiencing cardiovascular events vs. controls, only GDF-15 emerged statistically significant after the application of false discovery correction statistics. For the purposes of mechanistic insight, candidates for further validation, and potential value to others, details of the full panel are provided in Supplementary material online, *Table S5*. It is interesting to note that these cluster around inflammatory, renal, and extracellular matrix pathways.

Growth differentiation factor 15 was found to be significantly higher and demonstrated to be a candidate biomarker to predict cardiovascular events at 1 year [AUC of 0.715 (95% CI 0.63–0.81)], even after adjusting for clinical variables. What makes this a particularly remarkable and potentially important observation is that GDF-15 was initially discovered as a gene induced by NSAIDs (also known as *NAG-1*). Early experiments in 2001 by Baek *et al.*^16^ demonstrated that in colorectal cells, NSAIDs stimulated the expression of a transforming growth factor (TGF)-*β* superfamily protein that they named *NAG-1*. Growth differentiation factor 15 is a driver of cell growth arrest and apoptosis, which has since been hypothesized as a potential mechanism for the chemo-preventative effects of NSAIDs.^16^

While GDF-15 expression in tissues is low under normal physiological conditions, in pathological conditions, GDF-15 is produced by several cell types with ranging roles.^16^ In atherosclerotic models, GDF-15 has pro-inflammatory effects,^17,18^ whereas in acute myocardial infarction models in mice, GDF-15 has anti-inflammatory effects.^19^ Growth differentiation factor 15 is also expressed in response to hypoxia, inflammation, and oxidative stress. Interest in GDF-15 as a biomarker has increased at pace with just 7 publications using GDF-15 as a search term in PubMed in 2000 rising to 321 in 2023. There are now over 20 studies investigating its role as a cardiovascular biomarker.^20^

Most recently, a meta-analysis including a pool of over 50 000 patients reported that higher GDF-15 was associated with a significantly increased rate of cardiovascular death and MACE.^21^ These findings were also confirmed in a patient level meta-analysis including over 50 000 patients from 8 clinical trials. They found that in patients with atherosclerotic disease, higher GDF-15 levels were significantly and independently associated with increased rate of cardiovascular death, hospitalization for heart failure, and major adverse cardiac events. The authors concluded that GDF-15 is a useful cardiovascular biomarker, which is of additional prognostic value beyond established clinical risk factors and cardiac biomarkers.^21^

Our findings also support a role for GDF-15 measurements within a risk stratification tool for individuals taking NSAIDs long term. It is well established that NSAID use increases cardiovascular risk within populations by around 30%; however, the actual risk for an individual patient remains unknown. The PRECISION trialists in 2019 attempted to develop an NSAID major toxicity risk score,^22^ but this relied on a composite of both cardiovascular and renal endpoints in a high cardiovascular risk group. Given the widespread use of NSAIDs and the potential scale of the problem, the development of cardiovascular risk stratification tools in NSAID users should be re-evaluated, with aims of allowing informed decision-making when starting NSAIDs for both clinicians and patients, facilitating the re-introduction of celecoxib as chemo-preventative agents and allowing targeted use of preventative drugs such as statins.

While GDF-15 is a biomarker of cardiovascular disease and is also induced by NSAIDs, a direct link and/or cause and effect are unclear and outside the scope of the current study but remains the subject of investigation.

## Limitations

Data were prospectively derived from a randomized control trial with a low rate of missing data. While the aim of the study was to explore clinical drivers of cardiovascular events in NSAID users and not to develop a risk score, there are some important limitations that need to be acknowledged. There may be unknown drivers of cardiovascular events that have not been captured within the cohort. In this dataset, the primary outcome is relatively unbalanced with <4% of cases having the primary endpoint. This can affect the quality of any associations made. There were a high number of dropouts in the trial (51% celecoxib arm and 31% usual NSAID group). It is important to also highlight that this work was exploratory, and any statistical relationship does not imply causation. Results need to be replicated and validated in larger datasets or clinical trials. Finally, our results may not be generalizable as SCOT was a low cardiovascular risk population and >99% of the cohort were Caucasian.

All the patients in SCOT at screening were already on an NSAID. While we know ‘chronic NSAID user’ was an inclusion criterion, we do not know exactly how long participants were on an NSAID for or whether they had active NSAID in their system at the time of blood collection.

Though every effort has been taken to minimize confounding, this is still a case control study derived from a cohort of NSAID users, so unknown confounders may have not been considered. There is also a risk of overfitting when performing ROC or regression analysis. Finally, there is a relatively small sample size for the case–control study. As such, while these findings could have important clinical relevance, they need to be externally validated.

## Lead author biography


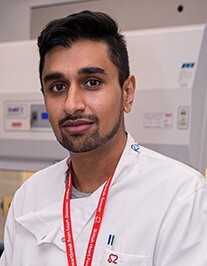


Ricky Vaja is a cardiothoracic surgeon currently training in London. He graduated from the University of Southampton in 2012 with a degree in medicine. He was awarded a BHF clinical research fellowship and completed a PhD focusing on biomarkers of COX-2 inhibition and the cardiovascular side effects of NSAIDs at Imperial College London. He is also actively involved in surgical trials and is the co-founder/lead of the Cardiothoracic Interdisciplinary Research Network.

## Supplementary Material

oeae088_Supplementary_Data

## Data Availability

Raw data generated for all circulating biomarkers will be made available upon request. The ownership of raw clinical data generated by the SCOT study lies with the SCOT investigators and so requests for clinical data need to be made directly to the SCOT investigators.
