# Supplementary material for: Vascular and inflammatory biomarkers of cardiovascular events in non-steroidal anti-inflammatory drug users
Source: Eur Heart J Open. 2024 Nov 2;4(6):oeae088. doi: 10.1093/ehjopen/oeae088 (PMC11630077; doi:10.1093/ehjopen/oeae088)
Supplement: oeae088_Supplementary_Data [file oeae088_supplementary_data.docx]

# **Supplementary file.**

**Title: Vascular and inflammatory biomarkers of cardiovascular events in non-steroidal anti-inflammatory drug (NSAID) users**

**Short Title: Biomarkers of cardiovascular events in NSAID users**

**Authors:** Ricky Vaja^1,3^, Plinio Ferreira^1^, Laura Portas^1^, Blerina Ahmetaj-Shala^1^, Neringa Cypaite^1^, Hime Gashaw^1^, Jennifer Quint^1^, Ramzi Khamis^1^, Adam Hartley^1^, Thomas M MacDonald^2^ , Isla S Mackenzie^2^, Nicholas S Kirkby^1^*, Jane A Mitchell^1^*

**Section 1: Statistical analysis of clinical predictors of cardiovascular events and Selection of candidate variables**

The intention to treat cohort was used for analysis. Cases were defined as participants who reached the primary end point during the study which included a composite of cardiovascular death, non-fatal stroke, non-fatal myocardial infarction or biomarker positive acute coronary syndrome or hospitalisation for non-fatal myocardial infarction. Controls were identified as participants who did not reach the primary endpoint. The methodology is presented in accordance with the Transparent Reporting of a Multivariable Prediction Model for Individual Prognosis or Diagnosis (TRIPOD) statement for developing and validating multivariable prediction models [1].

Clinical variables available within the dataset included age, gender, ethnicity, chronic obstructive airway disease (COPD), smoking status (current, ex-smoker and never smoked), rheumatoid arthritis, blood pressure, body mass index (BMI), Waist: hip ratio, diabetes, heart failure, total cholesterol, high density lipoprotein (LDL), triglycerides, renal disease, statins, aspirin, hypertension and uric acid. Analysis within the primary SCOT publication [2] showed that the type of NSAID was not associated with cardiovascular events and so was included as a variable in this analysis.

Causal relationships between variables in the dataset were visualised using direct acylic graphs. Four variables were excluded for the following reasons:

1. BMI- waist:hip ratio has been reported to be a better predictor of cardiovascular risk [3, 4]
2. COPD- COPD is often poorly and underdiagnosed, smoking status was deemed to be a more reliable predictor [3, 4]
3. Total cholesterol, HDL, triglycerides – Total cholesterol/HDL ratio >5 is a well-established predictor of cardiovascular risk
4. Hypertension- Data relating to blood pressure control and use of anti-hypertensives was not available. The average of three blood pressure readings at screening was judged to be a more reliable marker of uncontrolled hypertension. Blood pressure was categorised into <130mmHg, 130-159mmHg and ≥160mmHg[5].

Variables with less than 15 events per variable included non-Caucasian (n:1), heart failure (n:1) and renal disease (n:10) were not included in the analysis. There is debate as to the minimum number of events per variables [6], therefore a sensitivity analysis weas performed with the inclusion of renal disease which did not affect the model.

Candidate variables were selected using least absolute shrinkage and selection operator (LASSO).

# **Supplementary Table 1. Baseline characteristics Case control group**

| **Baseline Characteristic** | **n:97**  **No Event (n or Median)** | **% or IQR** | **n:49**  **Cardiovascular Events (n or Median)** | **% or IQR** |
| --- | --- | --- | --- | --- |
| Treatment Group | 52 | 53.61% | 30 | 61.22% |
| Control | 45 | 46.39% | 19 | 38.78% |
| *NSAID during Trial* |  |  |  |  |
| Ibuprofen | 23 | 23.71% | 8 | 16.33% |
| Diclofenac | 8 | 8.25% | 8 | 16.33% |
| Other NSAID | 14 | 14.43% | 3 | 6.12% |
| Celecoxib | 52 | 53.61% | 30 | 61.22% |
| Age (Years) | 67.02 | 8.51 | 67.54 | 10.96 |
| Age> 70 years | 36 | 37.11% | 19 | 38.78% |
| Age> 80 years | 4 | 4.12% | 4 | 8.16% |
| Male | 57 | 58.76% | 29 | 59.18% |
| Non-Caucasian | 0 | 0% | 0 | 0% |
| Current smoker | 16 | 16.49% | 17 | 34.69% |
| Never smoked | 42 | 43.30% | 7 | 14.29% |
| Previous smoker | 39 | 40.21% | 25 | 51.02% |
| Statin therapy | 15 | 15.46% | 7 | 14.29% |
| Aspirin therapy | 16 | 16.49% | 11 | 22.45% |
| Heart Failure | 0 | 0% | 1 | 2% |
| Hypertension | 46 | 47.42% | 16 | 32.65% |
| Systolic BP | 143 | 24.25 | 136.5 | 24.25 |
| Systolic BP> 140 | 52 | 53.61% | 20 | 40.82% |
| Systolic BP> 150 | 32 | 32.99% | 14 | 28.57% |
| Systolic BP> 160 | 20 | 20.62% | 8 | 16.33% |
| Diastolic BP | 80 | 15.25 | 75.5 | 14.5 |
| Diastolic BP> 100 | 1 | 1.03% | 1 | 2.04% |
| Hypercholesterolaemia | 35 | 36.08% | 6 | 12.24% |
| Renal Disease | 5 | 5.15% | 3 | 6.12% |
| Asthma | 22 | 22.68% | 5 | 10.20% |
| COPD | 7 | 7.22% | 2 | 4.08% |
| BMI | 29.05 | 6.28 | 27.34 | 5.36 |
| BMI < 18.5 | 0 | 0.00% | 0 | 0.00% |
| BMI>30 | 41 | 42.27% | 14 | 28.57% |
| Waist hip ratio | 0.94 | 0.1 | 0.92 | 0.13 |
| Waist hip ratio >1 | 20 | 20.62% | 11 | 22.45% |
| Total Cholesterol (mmol/l)** | 5.34 | 1.77 | 5.43 | 1.47 |
| Total Cholesterol >6** | 26 | 27.66% | 16 | 32.65% |
| HDL cholesterol** | 1.33 | 0.47 | 1.35 | 0.52 |
| HDL cholesterol<1** | 13 | 27.66% | 4 | 8.16% |
| Triglycerides (mmol/l)** | 1.64 | 1.07 | 1.68 | 1.11 |
| Total Chol:HDL ratio** | 3.99 | 1.3 | 3.89 | 1.71 |
| T:H Ratio >5** | 16 | 27.66% | 12 | 24.49% |
| Uric Acid | 329 | 125 | 331 | 139 |

***Supplementary Table 1. Baseline characteristics table comparing cases (cardiovascular event) and controls (no event).*** *Abbreviations used: BP: blood pressure, COPD: chronic obstructive pulmonary disease, BMI: body mass index, WHR: waist hip ratio. HDL: high density lipoprotein, chol: cholesterol. (** indicates n:94 controls, 49 events). Data is presented as n and % for dichotomous variables and median and IQR for continuous variables.*

**Section 2: Methodology for biomarker analysis**

**2.1: Cardiometabolic Proteomic analysis**

Targeted analysis of 369 cardiometabolic proteins was performed using Olink [7] which utilises Proximity Extension Assay (PEA) technology. The assays were performed by Olink laboratories, Uppsala Science Park, Sweden who were blinded to the groups. PCA analysis was performed using SIMCA 13.0.3 (Sartorius Stedim biotech, France) and differential expression was performed in IBM SPSS. Graphs for individual analytes were generated using GraphPad Prism.

**2.2: Methylarginine and amine quantification**

Quantification of amino acids and methylarginines was performed using a UHPLC-MS/MS platform that has previously been published [8]. In brief, 20μl serum was used and 5μl of internal standard (contains 16 stable-isotope-labelled internal standards & is used a quality check for extraction) was added. Protein was precipitated using 40μl of 0.1% formic acid and stored at -20 °C for 20 minutes and then centrifuged for 10 minutes at 10,000g. Next 10μl of the supernatant was transferred into another Eppendorf and 70μl of borate buffer was added, and samples were centrifuged at 2000g for 2 minutes. Sample derivatisation was performed using 20μl of 6-aminoquinolyl-N-hydroxysuccinimifyl carbamate (AccQTagTM kit - Waters, Wilmslow, UK). This maximises detection of amino acids and amines. Samples were heat sealed at 55°C for 10 minutes. Finally, the sample was diluted 1/10 using OptimaTM LC-MS grade water (Fisher Scientific; Leicester, UK). Samples were then loaded onto the UHPLC/MS/MS. Data acquired was then quantified using MassLynx V4.1 software (Waters, Milford, MA, USA) and concentrations extracted based on standards prepared and run along with samples.

**2.3: Enzyme-Linked Immunosorbent Assay**

*2.3.1: C-Reactive Protein*

CRP was measured using Human CRP duoset ELISA (Bio-techne R&D systems, Catalog #: DY1707.

2.3.2: *MDA-LDL*

ELISAs used to detect both MDA-LDL and anti-oxLDL antibodies were performed according to previously published methods [9, 10]. MDA-LDL was measured using LO1, a laboratory-developed monoclonal IgG3κ murine antibody, which acts as the capture antibody in a sandwich ELISA. Detection was performed using horseradish peroxidase (HRP)-conjugated streptavidin (R&D Systems, Minneapolis, MN, USA) at 1:200 dilution) and biotinylated anti-ApoB antibody (Abcam, Cambridge, MA, USA) at 1:2000 dilution. To this 3,3′,5,5′-tetramethylbenzidine (TMB) (Sigma Aldrich, Poole, UK) was added and the reaction stopped using 0.5 M H2SO4. Plates were read at an optical density (OD) of 450 nm using a Synergy HT microplate reader (BioTek, Winooski, VT, USA). Biotinylated mouse anti-human IgM (Cambridge Bioscience) or mouse anti-human IgG (Cambridge Bioscience, Cambridge, UK) followed by HRP-conjugated streptavidin (R&D Systems, Minneapolis, MN, USA) or HRP-conjugated rabbit anti-mouse Ig (Dako, Cambridgeshire, UK) were used to detect IgG and IgM antibody binding to solid phase antigens in an Indirect ELISA format. No standard curve is used for this method and data was generated as raw OD values. Data was therefore presented as Z-score.

*Total IgG and IgM Antibodies*

As described previously, an in-house ELISA was used consisting of mouse anti-human IgM or goat anti-human IgG (Southern Biotech, Birmingham, AL, USA). Biotinylated goat F(ab′)² anti-human IgG or biotinylated mouse anti-human IgM (both Southern Biotech) were used as detection antibodies.

# **Supplementary Table 2. Baseline characteristics overall SCOT cohort**

| **Baseline Characteristic** | **n: 7048** | **% or IQR** | **n: 249** | **% or IQR** |
| --- | --- | --- | --- | --- |
|  | **No Event (n or Median)** |  | **Cardiovascular Events (n or Median)** |  |
| Treatment Group (Celecoxib) | 3522 | 49.97% | 125 | 50.20% |
| Control (usual NSAID) | 3526 | 50.03% | 124 | 49.80% |
| *Country* |  |  |  |  |
| Scotland | 3817 | 54.20% | 150 | 60.24% |
| Denmark | 2135 | 30.30% | 74 | 29.72% |
| England | 1053 | 14.90% | 25 | 10.04% |
| Netherlands | 43 | 0.60% | 0 | 0% |
| *NSAID during Trial* |  |  |  |  |
| Ibuprofen | 1114 | 15.80% | 39 | 15.66% |
| Diclofenac | 1359 | 19.30% | 53 | 21.29% |
| Other NSAID | 1053 | 14.90% | 32 | 12.85% |
| Celecoxib | 3522 | 50% | 125 | 50.20% |
| Age (Years) | 67 | 8.8 | 69.4 | 10.4 |
| Age 60-70 | 4615 | 65.48% | 130 | 52.20% |
| Age 70-80 | 2091 | 29.70% | 86 | 34.50% |
| Age> 80 | 342 | 4.90% | 33 | 13.25% |
| Male | 2818 | 40% | 141 | 56.63% |
| Non-Caucasian | 22 | 0.30% | 1 | 0.40% |
| Current Smoker | 1063 | 15.10% | 67 | 26.91% |
| Never smoker | 2929 | 41.60% | 71 | 28.51% |
| Rheumatoid Arthritis | 434 | 6.20% | 20 | 8.03% |
| Statin therapy | 1484 | 21.10% | 38 | 15.26% |
| Aspirin therapy | 810 | 11.50% | 44 | 17.67% |
| Heart Failure | 13 | 0.20% | 1 | 0.40% |
| History of Diabetes | 559 | 7.90% | 27 | 10.84% |
| Hypertension | 3136 | 44.50% | 104 | 41.77% |
| Systolic BP * | 139.5 | 22.5 | 140.5 | 24.5 |
| Systolic BP < 130 mmHg * | 1943 | 27.58% | 59 | 23.69% |
| Systolic BP 130 – 160mmHg * | 4178 | 59.31% | 145 | 58.23% |
| Systolic BP> 160mmHg * | 893 | 12.70% | 45 | 17.67% |
| Diastolic BP * | 77 | 14 | 75.5 | 15 |
| Diastolic BP> 100mmHg * | 163 | 2.30% | 6 | 2.41% |
| Renal Disease | 248 | 3.50% | 10 | 4.02% |
| Asthma | 699 | 9.90% | 28 | 11.24% |
| COPD | 296 | 4.20% | 21 | 8.43% |
| BMI | 28.7 | 6.8 | 28.5 | 6.7 |
| Waist hip ratio | 0.91 | 0.1 | 0.93 | 0.1 |
| WHR >1 | 1012 | 14.40% | 50 | 20.08% |
| Hypercholesterolaemia | 2421 | 34.40% | 58 | 23.29% |
| Total Cholesterol (mmol/l)** | 5.23 | 1.48 | 5.31 | 1.59 |
| Total cholesterol > 5.5** | 2501 | 39.70% | 97 | 42.92% |
| HDL cholesterol* | 1.38 | 0.51 | 1.29 | 0.42 |
| HDL<1 | 655 | 10.40% | 33 | 14.60% |
| Triglycerides ** | 1.63 | 1.1 | 1.68 | 1.1 |
| Tot Chol:HDL Ratio** | 3.71 | 1.33 | 4 | 1.5 |
| T:H Ratio >5 ** | 773 | 12.27% | 49 | 21.68% |
| Uric Acid (mmol/l)*** | 319 | 110 | 327 | 125 |
| Uric Acid<300 umol/l*** | 2609 | 40.62% | 82 | 35.96% |
| Uric Acid 300-400 umol/l*** | 2628 | 40.92% | 92 | 40.35% |
| Uric Acid>400 umol/l*** | 1186 | 18.46% | 54 | 23.68% |

***Supplementary Table 2. Baseline characteristics table comparing cases (cardiovascular event) and controls (no event).*** *Abbreviations used:*

*BP: blood pressure, COPD: chronic obstructive pulmonary disease, BMI: body mass index, WHR: waist hip ratio. HDL: high density lipoprotein, chol: cholesterol.*

**n: 7044 in non-event group and 249 in event group.*

***n:6300 in the non-event group and 226 in the event group.*

****n: 6423 in non-event group and 228 in event group.*

*Data is presented as n and % for dichotomous variables and median and IQR for continuous variables.*

# .

**Supplementary Table 3. Factors associated with a cardiovascular event using Binary logistic regression- stratified to NSAID used.**

|  | **Celecoxib (3149 controls, 111 events)** | | | | **Ibuprofen (1016 controls, 37 events)** | | | | **Diclofenac (1192 controls, 48 events)** | | | | **Other NSAID (941 controls, 30 events)** | | | |
| --- | --- | --- | --- | --- | --- | --- | --- | --- | --- | --- | --- | --- | --- | --- | --- | --- |
| **Variable** | **OR** | **95% C.I. for OR** | | **p value** | **OR** | **95% C.I. for OR** | | **p value** | **OR** | **95% C.I. for OR** | | **p value** | **OR** | **95% C.I. for OR** | | **p value** |
|  |  | **Lower** | **Upper** |  |  | **Lower** | **Upper** |  |  | **Lower** | **Upper** |  |  | **Lower** | **Upper** |  |
| **Sex** |  |  |  |  |  |  |  |  |  |  |  |  |  |  |  |  |
| Female |  |  |  |  |  |  |  |  |  |  |  |  |  |  |  |  |
| Male | 1.65 | 1.06 | 2.55 | 0.026 | 1.92 | 0.87 | 4.24 | 0.108 | 1.91 | 0.99 | 3.7 | 0.056 | 1.72 | 0.71 | 4.15 | 0.229 |
| **Smoking Status** |  |  |  |  |  |  |  |  |  |  |  |  |  |  |  |  |
| Non-Smoker |  |  |  |  |  |  |  |  |  |  |  |  |  |  |  |  |
| Current Smoker | 2.56 | 1.52 | 4.32 | <0.0001 | 2.6 | 0.92 | 7.37 | 0.071 | 2.53 | 1.07 | 5.99 | 0.034 | 1.87 | 0.73 | 4.77 | 0.19 |
| Former smoker | 1.19 | 0.75 | 1.9 | 0.46 | 1.96 | 0.81 | 4.73 | 0.137 | 1.18 | 0.85 | 3.8 | 0.128 | 0.64 | 0.25 | 1.59 | 0.33 |
| **Systolic blood pressure at screening** |  |  |  |  |  |  |  |  |  |  |  |  |  |  |  |  |
| < 130mmHg |  |  |  |  |  |  |  |  |  |  |  |  |  |  |  |  |
| 130 – 159 mmHg | 1.28 | 0.78 | 2.09 | 0.325 | 1.31 | 0.49 | 3.45 | 0.59 | 0.89 | 0.46 | 1.71 | 0.718 | 0.68 | 0.29 | 1.58 | 0.371 |
| ≥160mmHg | 1.47 | 0.77 | 2.83 | 0.244 | 4.4 | 1.6 | 12.08 | 0.004 | 0.73 | 0.27 | 1.98 | 0.53 | 0.73 | 0.21 | 2.53 | 0.62 |
| **Arthritis** |  |  |  |  |  |  |  |  |  |  |  |  |  |  |  |  |
| Osteoarthritis |  |  |  |  |  |  |  |  |  |  |  |  |  |  |  |  |
| Rheumatoid arthritis | 1.34 | 0.63 | 2.84 | 0.447 | 2.16 | 0.58 | 8.1 | 0.25 | 3.27 | 1.16 | 9.21 | 0.025 | 1.24 | 0.35 | 4.37 | 0.74 |
| **Diabetes** |  |  |  |  |  |  |  |  |  |  |  |  |  |  |  |  |
| No |  |  |  |  |  |  |  |  |  |  |  |  |  |  |  |  |
| Yes | 1.78 | 0.94 | 3.38 | 0.76 | 1.16 | 0.32 | 4.15 | 0.822 | 1.03 | 0.334 | 3.17 | 0.959 | 1.99 | 0.71 | 5.57 | 0.19 |
| **Concomitant aspirin use** |  |  |  |  |  |  |  |  |  |  |  |  |  |  |  |  |
| No |  |  |  |  |  |  |  |  |  |  |  |  |  |  |  |  |
| Yes | 1.6 | 0.95 | 2.69 | 0.079 | 1.2 | 0.47 | 3.12 | 0.7 | 1.42 | 0.64 | 3.13 | 0.384 | 1.76 | 0.65 | 4.8 | 0.266 |
| **Total cholesterol : HDL ratio** |  |  |  |  |  |  |  |  |  |  |  |  |  |  |  |  |
| <5 |  |  |  |  |  |  |  |  |  |  |  |  |  |  |  |  |
| ≥5 | 1.92 | 1.16 | 3.17 | 0.011 | 1.7 | 0.7 | 4.34 | 0.232 | 2.31 | 1.16 | 4.58 | 0.017 | 1.57 | 0.6 | 4.15 | 0.36 |
| **Age** |  |  |  |  |  |  |  |  |  |  |  |  |  |  |  |  |
| 60-69 years |  |  |  |  |  |  |  |  |  |  |  |  |  |  |  |  |
| 70-79 Years | 1.79 | 1.17 | 2.75 | 0.007 | 1.48 | 0.68 | 3.2 | 0.321 | 1.83 | 0.96 | 3.47 | 0.065 | 1.23 | 0.52 | 2.92 | 0.64 |
|  |  |  |  |  |  |  |  |  |  |  |  |  |  |  |  |  |
| ≥80 years | 4.61 | 2.56 | 8.3 | <0.001 | 5.34 | 2.01 | 14.22 | 0.001 | 2.44 | 0.67 | 8.88 | 0.175 | 3.69 | 0.95 | 14.3 | 0.059 |
| **Statin Use** |  |  |  |  |  |  |  |  |  |  |  |  |  |  |  |  |
| No |  |  |  |  |  |  |  |  |  |  |  |  |  |  |  |  |
| Yes | 0.417 | 0.23 | 0.78 | 0.006 | 0.35 | 0.1 | 1.21 | 0.097 | 1.44 | 0.72 | 2.88 | 0.308 | 0.92 | 0.36 | 2.38 | 0.86 |
| **Uric acid** |  |  |  |  |  |  |  |  |  |  |  |  |  |  |  |  |
| Uric acid <300umol/l |  |  |  |  |  |  |  |  |  |  |  |  |  |  |  |  |
| Uric acid 300 - 400umol/l | 1.02 | 0.65 | 1.59 | 0.936 | 0.73 | 0.31 | 1.74 | 0.479 | 0.84 | 0.42 | 1.71 | 0.635 | 0.92 | 0.38 | 2.2 | 0.842 |
| Uric acid >400umol/l |  |  |  |  | 1.41 | 0.58 | 3.42 | 0.452 | 1.36 | 0.59 | 3.11 | 0.47 | 1.19 | 0.42 | 3.39 | 0.741 |
| **Waist: Hip ratio** |  |  |  |  |  |  |  |  |  |  |  |  |  |  |  |  |
| <1 |  |  |  |  |  |  |  |  |  |  |  |  |  |  |  |  |
| ≥1 | 1.04 | 0.62 | 1.75 | 0.876 | 1.08 | 0.44 | 2.63 | 0.87 | 0.4 | 0.15 | 1.1 | 0.075 | 2.84 | 1.17 | 6.88 | 0.021 |

***Supplementary Table 3.*** *Factors associated with a cardiovascular event using binary logistic regression stratified to NSAID used. The model includes all variables listed (Male gender, smoking status, systolic Blood pressure, arthritis, diabetes, waist: hip ratio, statin use, aspirin use, total cholesterol: HDL ratio, uric acid and age. Abbreviations: SE: standard error, OR: odds ratio, CI: confidence interval. Each variable is adjusted for all other variables shown.*

# **Supplementary Table 4. Concentrations of methylarginines and amines**

| **Analyte** | **Group** | **N** | **Mean** | **Std. Deviation** | **Std. Error Mean** |
| --- | --- | --- | --- | --- | --- |
| 4-hydroxyproline | Event | 49 | 7.14 | 3.09 | 0.44 |
|  | Control | 97 | 7.53 | 3.60 | 0.37 |
| alanine | Event | 49 | 324.02 | 108.19 | 15.46 |
|  | Control | 97 | 313.18 | 111.77 | 11.35 |
| Arginine | Event | 49 | 81.21 | 32.40 | 4.63 |
|  | Control | 97 | 82.61 | 33.80 | 3.43 |
| Aspartic acid | Event | 49 | 30.71 | 16.87 | 2.41 |
|  | Control | 97 | 27.28 | 10.71 | 1.09 |
| Asparagine | Event | 49 | 29.27 | 9.57 | 1.37 |
|  | Control | 97 | 27.43 | 9.44 | 0.96 |
| Cystine | Event | 49 | 12.68 | 8.99 | 1.28 |
|  | Control | 97 | 12.20 | 11.22 | 1.14 |
| Ethanolamine | Event | 49 | 7.79 | 3.00 | 0.43 |
|  | Control | 97 | 7.25 | 2.51 | 0.25 |
| Glutamic acid | Event | 49 | 109.64 | 54.29 | 7.76 |
|  | Control | 97 | 99.44 | 40.77 | 4.14 |
| Glutamine | Event | 49 | 229.99 | 67.57 | 9.65 |
|  | Control | 97 | 220.33 | 71.98 | 7.31 |
| Glycine | Event | 49 | 127.83 | 37.58 | 5.37 |
|  | Control | 97 | 123.54 | 26.55 | 2.70 |
| Histidine | Event | 49 | 55.88 | 22.02 | 3.15 |
|  | Control | 97 | 53.80 | 18.33 | 1.86 |
| Isoleucine | Event | 49 | 37.92 | 14.94 | 2.13 |
|  | Control | 97 | 39.50 | 16.49 | 1.67 |
| Leucine | Event | 49 | 47.49 | 13.23 | 1.89 |
|  | Control | 97 | 48.44 | 12.39 | 1.26 |
| Lysine 244 | Event | 49 | 84.27 | 22.29 | 3.18 |
|  | Control | 97 | 86.40 | 23.36 | 2.37 |
| Methionine | Event | 49 | 14.80 | 5.95 | 0.85 |
|  | Control | 97 | 14.44 | 6.62 | 0.67 |
| Phenylalanine | Event | 49 | 49.67 | 13.60 | 1.94 |
|  | Control | 97 | 49.25 | 12.70 | 1.29 |
| Proline | Event | 49 | 160.62 | 63.35 | 9.05 |
|  | Control | 97 | 154.40 | 65.56 | 6.66 |
| Serine | Event | 49 | 94.15 | 33.82 | 4.83 |
|  | Control | 97 | 93.19 | 25.75 | 2.61 |
| Threonine | Event | 49 | 58.20 | 17.29 | 2.47 |
|  | Control | 97 | 56.96 | 14.25 | 1.45 |
| Tryptophan | Event | 49 | 44.63 | 18.24 | 2.61 |
|  | Control | 97 | 47.61 | 16.01 | 1.63 |
| Tyrosine | Event | 49 | 50.78 | 17.08 | 2.44 |
|  | Control | 97 | 50.85 | 15.45 | 1.57 |
| Valine | Event | 49 | 62.62 | 14.74 | 2.11 |
|  | Control | 97 | 62.89 | 13.93 | 1.41 |
| Beta-amino-iso-butyric acid | Event | 49 | 4.97 | 4.54 | 0.65 |
|  | Control | 97 | 4.88 | 4.58 | 0.47 |
| Citrulline | Event | 49 | 24.73 | 15.35 | 2.19 |
|  | Control | 97 | 22.09 | 8.69 | 0.88 |
| Cystathione | Event | 49 | 0.36 | 0.21 | 0.03 |
|  | Control | 97 | 0.40 | 0.31 | 0.03 |
| 3-methylhystidine | Event | 49 | 2.66 | 3.82 | 0.55 |
|  | Control | 97 | 2.92 | 6.03 | 0.61 |
| 1-methylhystidine | Event | 49 | 2.27 | 1.05 | 0.15 |
|  | Control | 97 | 2.25 | 1.27 | 0.13 |
| Ornithine | Event | 49 | 64.07 | 24.69 | 3.53 |
|  | Control | 97 | 65.88 | 25.41 | 2.58 |
| Aminoadipic acid | Event | 49 | 1.08 | 0.60 | 0.09 |
|  | Control | 97 | 1.15 | 0.59 | 0.06 |
| Alpha-amino-n-butyric acid | Event | 49 | 9.69 | 4.35 | 0.62 |
|  | Control | 97 | 10.49 | 4.03 | 0.41 |
| Taurine | Event | 49 | 67.37 | 36.45 | 5.21 |
|  | Control | 97 | 67.66 | 34.75 | 3.53 |
| beta-alanine | Event | 49 | 122.65 | 29.61 | 4.23 |
|  | Control | 97 | 120.96 | 26.43 | 2.68 |
| LNMMA | Event | 49 | 0.15 | 0.03 | 0.00 |
|  | Control | 97 | 0.15 | 0.03 | 0.00 |
| ADMA | Event | 49 | 0.78 | 0.27 | 0.04 |
|  | Control | 97 | 0.69 | 0.20 | 0.02 |
| SDMA | Event | 49 | 0.63 | 0.20 | 0.03 |
|  | Control | 97 | 0.58 | 0.22 | 0.02 |
| L-homoarginine | Event | 49 | 1.90 | 1.03 | 0.15 |
|  | Control | 97 | 1.77 | 1.00 | 0.10 |
| Arg/ADMA | Event | 49 | 107.64 | 33.18 | 4.74 |
|  | Control | 97 | 121.05 | 36.12 | 3.67 |
| GABR | Event | 49 | 0.98 | 0.35 | 0.05 |
|  | Control | 97 | 1.00 | 0.36 | 0.04 |

# **Supplementary Table 5. Proteomic data**

|  |  |  | **95% Confidence Interval** | |
| --- | --- | --- | --- | --- |
| **Protein** | **P value** | **Fold Change** | **Lower** | **Upper** |
| GDF15 | <0.001 | 1.353 | 1.185 | 1.546 |
| CHI3L1 | 0.001 | 1.366 | 1.129 | 1.653 |
| SSC4D | 0.002 | 0.569 | 0.398 | 0.815 |
| MMP7 | 0.006 | 1.070 | 1.020 | 1.124 |
| CST3 | 0.008 | 1.138 | 1.036 | 1.250 |
| CTSB | 0.009 | 1.221 | 1.053 | 1.416 |
| REG1A | 0.010 | 1.187 | 1.042 | 1.353 |
| FUCA1 | 0.011 | 1.329 | 1.068 | 1.653 |
| PAG1 | 0.012 | 1.323 | 1.065 | 1.644 |
| IGFBP7 | 0.013 | 1.143 | 1.029 | 1.269 |
| REG1B | 0.014 | 1.217 | 1.042 | 1.423 |
| COL18A1 | 0.014 | 1.094 | 1.018 | 1.175 |
| CD59 | 0.016 | 1.094 | 1.017 | 1.176 |
| PGLYRP1 | 0.017 | 1.212 | 1.035 | 1.420 |
| TNC | 0.018 | 1.171 | 1.028 | 1.335 |
| PTGDS | 0.020 | 1.108 | 1.016 | 1.209 |
| COL1A1 | 0.020 | 0.889 | 0.805 | 0.982 |
| ENTPD6 | 0.021 | 1.093 | 1.014 | 1.179 |
| CD14 | 0.022 | 1.154 | 1.021 | 1.304 |
| TFF3 | 0.026 | 1.158 | 1.018 | 1.318 |
| MSMB | 0.026 | 1.244 | 1.026 | 1.509 |
| ANGPTL3 | 0.028 | 1.126 | 1.013 | 1.252 |
| KITLG | 0.028 | 0.843 | 0.723 | 0.981 |
| TGFBR3 | 0.029 | 1.231 | 1.022 | 1.483 |
| CCL15 | 0.030 | 1.143 | 1.013 | 1.289 |
| NTproBNP | 0.033 | 1.536 | 1.035 | 2.281 |
| EGFR | 0.036 | 0.943 | 0.892 | 0.996 |
| IGFBP1 | 0.038 | 1.412 | 1.020 | 1.954 |
| IL2RA | 0.040 | 1.131 | 1.006 | 1.272 |
| REG3A | 0.041 | 1.248 | 1.010 | 1.542 |
| PRTN3 | 0.042 | 1.259 | 1.009 | 1.570 |
| LBP | 0.046 | 1.155 | 1.003 | 1.331 |
| CSTB | 0.047 | 1.208 | 1.003 | 1.456 |
| ACOX1 | 0.048 | 1.193 | 1.002 | 1.421 |
| COL6A3 | 0.056 | 1.115 | 0.997 | 1.246 |
| ACP5 | 0.057 | 1.154 | 0.996 | 1.338 |
| SERPINA11 | 0.057 | 1.132 | 0.996 | 1.286 |
| AOC3 | 0.059 | 1.083 | 0.997 | 1.176 |
| TSLP | 0.059 | 1.226 | 0.992 | 1.515 |
| RNASET2 | 0.060 | 1.076 | 0.997 | 1.161 |
| STK4 | 0.061 | 1.188 | 0.992 | 1.423 |
| TYMP | 0.062 | 1.144 | 0.993 | 1.317 |
| PTPRF | 0.066 | 0.925 | 0.852 | 1.005 |
| IL6R | 0.066 | 0.912 | 0.826 | 1.006 |
| HNRNPK | 0.067 | 1.232 | 0.985 | 1.542 |
| LEP | 0.069 | 0.730 | 0.520 | 1.026 |
| NTRK2 | 0.073 | 0.902 | 0.806 | 1.010 |
| CA5A | 0.077 | 1.282 | 0.973 | 1.690 |
| SDC4 | 0.078 | 0.843 | 0.697 | 1.020 |
| GYS1 | 0.080 | 1.390 | 0.961 | 2.010 |
| LCN2 | 0.081 | 1.123 | 0.986 | 1.280 |
| TYRO3 | 0.082 | 0.898 | 0.796 | 1.014 |
| DUOX2 | 0.087 | 0.833 | 0.676 | 1.027 |
| GRK5 | 0.087 | 1.115 | 0.984 | 1.263 |
| TIA1 | 0.088 | 1.139 | 0.980 | 1.324 |
| DEFA1_DEFA1B | 0.089 | 1.192 | 0.973 | 1.461 |
| BMP6 | 0.093 | 1.291 | 0.958 | 1.739 |
| AZU1 | 0.096 | 1.288 | 0.956 | 1.736 |
| CEBPB | 0.096 | 1.260 | 0.959 | 1.656 |
| MEP1B | 0.097 | 0.747 | 0.529 | 1.055 |
| PPIB | 0.100 | 1.160 | 0.972 | 1.385 |
| COMT | 0.102 | 1.316 | 0.946 | 1.829 |
| PRSS27 | 0.104 | 0.892 | 0.776 | 1.024 |
| CCN3 | 0.108 | 1.086 | 0.982 | 1.201 |
| IRAG2 | 0.108 | 1.247 | 0.952 | 1.634 |
| FCGR2A | 0.109 | 1.145 | 0.970 | 1.352 |
| OSMR | 0.113 | 1.049 | 0.989 | 1.113 |
| CDHR5 | 0.114 | 0.881 | 0.752 | 1.031 |
| VSTM2L | 0.114 | 1.135 | 0.970 | 1.329 |
| ITGB1BP2 | 0.114 | 1.247 | 0.948 | 1.642 |
| CCL14 | 0.115 | 1.068 | 0.984 | 1.159 |
| LILRB2 | 0.115 | 1.081 | 0.981 | 1.192 |
| PPP1R2 | 0.116 | 1.172 | 0.961 | 1.429 |
| MET | 0.116 | 0.960 | 0.913 | 1.010 |
| ACAN | 0.116 | 0.917 | 0.823 | 1.022 |
| ZBTB17 | 0.124 | 1.172 | 0.957 | 1.437 |
| NPPB | 0.124 | 1.437 | 0.904 | 2.285 |
| ADAMTS16 | 0.125 | 1.069 | 0.981 | 1.165 |
| CANT1 | 0.127 | 0.895 | 0.776 | 1.032 |
| SORT1 | 0.128 | 0.868 | 0.724 | 1.042 |
| CD46 | 0.128 | 1.072 | 0.980 | 1.172 |
| SUSD1 | 0.134 | 1.125 | 0.964 | 1.313 |
| TINAGL1 | 0.135 | 0.896 | 0.775 | 1.035 |
| RARRES2 | 0.137 | 1.084 | 0.974 | 1.206 |
| CHEK2 | 0.139 | 1.085 | 0.973 | 1.210 |
| CTSZ | 0.142 | 1.083 | 0.973 | 1.204 |
| TIMP1 | 0.142 | 1.051 | 0.983 | 1.123 |
| HSPG2 | 0.143 | 1.063 | 0.979 | 1.154 |
| ADAMTS13 | 0.146 | 0.940 | 0.865 | 1.022 |
| GUSB | 0.154 | 1.112 | 0.961 | 1.288 |
| IGFBP2 | 0.155 | 1.138 | 0.952 | 1.361 |
| CD209 | 0.156 | 1.080 | 0.971 | 1.202 |
| CNDP1 | 0.157 | 0.911 | 0.799 | 1.037 |
| STK11 | 0.158 | 1.229 | 0.922 | 1.639 |
| WASF1 | 0.159 | 1.138 | 0.950 | 1.362 |
| DDC | 0.159 | 0.867 | 0.710 | 1.058 |
| HK2 | 0.165 | 1.079 | 0.969 | 1.201 |
| VWF | 0.167 | 1.103 | 0.959 | 1.268 |
| BOC | 0.173 | 1.058 | 0.975 | 1.148 |
| ADGRG2 | 0.177 | 0.909 | 0.792 | 1.044 |
| TNFRSF10C | 0.180 | 1.093 | 0.959 | 1.246 |
| RCOR1 | 0.183 | 1.115 | 0.949 | 1.309 |
| CCL5 | 0.187 | 1.142 | 0.937 | 1.392 |
| CD93 | 0.198 | 1.053 | 0.973 | 1.140 |
| EPHB4 | 0.199 | 1.053 | 0.973 | 1.140 |
| CXCL16 | 0.200 | 1.052 | 0.973 | 1.136 |
| TCN2 | 0.203 | 1.057 | 0.970 | 1.151 |
| ALCAM | 0.207 | 1.042 | 0.977 | 1.112 |
| FAS | 0.207 | 1.102 | 0.947 | 1.284 |
| TNFSF13B | 0.210 | 1.054 | 0.970 | 1.146 |
| NOTCH3 | 0.211 | 0.938 | 0.848 | 1.038 |
| MARCO | 0.212 | 0.910 | 0.785 | 1.056 |
| SELE | 0.212 | 0.903 | 0.769 | 1.060 |
| ACTA2 | 0.215 | 1.094 | 0.949 | 1.262 |
| GPNMB | 0.216 | 0.932 | 0.833 | 1.043 |
| THBD | 0.216 | 0.899 | 0.760 | 1.065 |
| GAS6 | 0.222 | 1.046 | 0.973 | 1.124 |
| IL6 | 0.227 | 1.193 | 0.895 | 1.592 |
| SPARCL1 | 0.229 | 1.057 | 0.965 | 1.157 |
| EIF4EBP1 | 0.231 | 1.295 | 0.847 | 1.980 |
| GDF2 | 0.234 | 0.902 | 0.761 | 1.070 |
| DCN | 0.236 | 1.043 | 0.972 | 1.120 |
| IL6ST | 0.237 | 0.940 | 0.849 | 1.042 |
| NPTXR | 0.238 | 0.901 | 0.756 | 1.072 |
| LILRB1 | 0.246 | 1.054 | 0.964 | 1.152 |
| HYOU1 | 0.246 | 1.037 | 0.975 | 1.102 |
| CTSH | 0.248 | 0.861 | 0.666 | 1.112 |
| S100A11 | 0.252 | 1.102 | 0.933 | 1.301 |
| ENTPD5 | 0.253 | 1.033 | 0.977 | 1.092 |
| LDLR | 0.255 | 0.913 | 0.780 | 1.068 |
| PRSS2 | 0.258 | 1.091 | 0.937 | 1.270 |
| TGFBI | 0.261 | 0.953 | 0.877 | 1.037 |
| DPP4 | 0.266 | 0.952 | 0.874 | 1.038 |
| TNF | 0.269 | 1.110 | 0.922 | 1.338 |
| SOST | 0.273 | 0.905 | 0.757 | 1.082 |
| PTN | 0.274 | 1.135 | 0.903 | 1.427 |
| THBS4 | 0.275 | 0.919 | 0.788 | 1.071 |
| CORO1A | 0.276 | 1.211 | 0.857 | 1.712 |
| MCFD2 | 0.276 | 0.942 | 0.845 | 1.050 |
| TFPI | 0.277 | 1.042 | 0.967 | 1.121 |
| LGALS3 | 0.278 | 1.072 | 0.945 | 1.217 |
| NRCAM | 0.278 | 0.912 | 0.771 | 1.078 |
| TSPAN1 | 0.280 | 0.893 | 0.727 | 1.097 |
| FADD | 0.283 | 1.156 | 0.886 | 1.507 |
| DNAJB8 | 0.287 | 1.117 | 0.910 | 1.371 |
| GZMH | 0.288 | 1.210 | 0.850 | 1.724 |
| DPP7 | 0.289 | 1.092 | 0.927 | 1.286 |
| CRTAC1 | 0.291 | 0.957 | 0.883 | 1.038 |
| GLRX | 0.293 | 1.139 | 0.893 | 1.453 |
| BLMH | 0.296 | 1.081 | 0.934 | 1.250 |
| CASP3 | 0.298 | 1.171 | 0.869 | 1.578 |
| DKK3 | 0.299 | 1.055 | 0.953 | 1.167 |
| TNNI3 | 0.304 | 1.236 | 0.824 | 1.854 |
| XG | 0.304 | 1.085 | 0.928 | 1.270 |
| IL18BP | 0.306 | 1.044 | 0.961 | 1.135 |
| PLXNB2 | 0.310 | 0.965 | 0.901 | 1.034 |
| COMP | 0.312 | 0.947 | 0.853 | 1.053 |
| GSTA1 | 0.313 | 0.874 | 0.673 | 1.136 |
| LACTB2 | 0.321 | 1.131 | 0.885 | 1.446 |
| PM20D1 | 0.322 | 0.785 | 0.486 | 1.270 |
| CLUL1 | 0.323 | 0.925 | 0.791 | 1.081 |
| SNX9 | 0.324 | 1.155 | 0.866 | 1.539 |
| FBP1 | 0.324 | 1.151 | 0.869 | 1.526 |
| PAM | 0.325 | 1.039 | 0.962 | 1.122 |
| ICAM2 | 0.326 | 1.042 | 0.959 | 1.132 |
| ICAM1 | 0.327 | 1.043 | 0.959 | 1.134 |
| LRP11 | 0.328 | 0.926 | 0.793 | 1.081 |
| NCAM1 | 0.333 | 0.959 | 0.882 | 1.044 |
| PILRB | 0.333 | 1.091 | 0.914 | 1.302 |
| CDH2 | 0.335 | 0.932 | 0.808 | 1.076 |
| ENG | 0.338 | 1.024 | 0.975 | 1.076 |
| MPHOSPH8 | 0.338 | 1.118 | 0.889 | 1.405 |
| AMY2B | 0.339 | 0.933 | 0.809 | 1.076 |
| CTSL | 0.344 | 0.952 | 0.860 | 1.054 |
| QPCT | 0.346 | 1.036 | 0.962 | 1.115 |
| CDH17 | 0.349 | 0.917 | 0.765 | 1.100 |
| TGM2 | 0.354 | 1.172 | 0.837 | 1.641 |
| LILRA5 | 0.355 | 0.932 | 0.803 | 1.082 |
| SIRPA | 0.356 | 0.937 | 0.816 | 1.077 |
| CA4 | 0.359 | 1.037 | 0.959 | 1.123 |
| FAP | 0.360 | 0.963 | 0.887 | 1.045 |
| PRCP | 0.362 | 1.067 | 0.928 | 1.226 |
| UMOD | 0.368 | 0.933 | 0.801 | 1.086 |
| CRX | 0.368 | 1.140 | 0.856 | 1.519 |
| IGFBP3 | 0.369 | 0.952 | 0.855 | 1.061 |
| THPO | 0.375 | 1.046 | 0.946 | 1.157 |
| SCARF1 | 0.376 | 1.056 | 0.935 | 1.193 |
| ESAM | 0.377 | 1.034 | 0.959 | 1.115 |
| AXL | 0.378 | 0.969 | 0.905 | 1.039 |
| CA3 | 0.378 | 1.131 | 0.859 | 1.489 |
| MSTN | 0.378 | 0.930 | 0.789 | 1.095 |
| FABP2 | 0.382 | 1.122 | 0.865 | 1.455 |
| CCL18 | 0.389 | 1.069 | 0.918 | 1.245 |
| NPDC1 | 0.391 | 1.074 | 0.912 | 1.265 |
| PDGFRB | 0.396 | 0.955 | 0.858 | 1.063 |
| CCL27 | 0.399 | 1.073 | 0.910 | 1.266 |
| IGFBP6 | 0.404 | 1.044 | 0.943 | 1.157 |
| TIE1 | 0.405 | 1.047 | 0.940 | 1.165 |
| TSHB | 0.408 | 1.098 | 0.879 | 1.373 |
| LGALS1 | 0.414 | 1.061 | 0.920 | 1.223 |
| EFEMP1 | 0.423 | 1.042 | 0.942 | 1.152 |
| MFAP3 | 0.424 | 0.919 | 0.746 | 1.132 |
| PI3 | 0.425 | 1.063 | 0.914 | 1.236 |
| C1QTNF1 | 0.429 | 1.037 | 0.947 | 1.136 |
| MTPN | 0.432 | 1.042 | 0.940 | 1.156 |
| DIABLO | 0.436 | 1.115 | 0.847 | 1.467 |
| TIMD4 | 0.446 | 1.046 | 0.931 | 1.176 |
| VAMP5 | 0.450 | 1.084 | 0.878 | 1.339 |
| CDH1 | 0.450 | 0.969 | 0.894 | 1.051 |
| NADK | 0.451 | 1.114 | 0.840 | 1.478 |
| PDGFA | 0.463 | 0.958 | 0.852 | 1.076 |
| APLP1 | 0.464 | 0.946 | 0.816 | 1.098 |
| ADA2 | 0.466 | 1.046 | 0.926 | 1.183 |
| ADAM15 | 0.467 | 1.036 | 0.941 | 1.141 |
| AMY2A | 0.468 | 0.950 | 0.827 | 1.091 |
| ART3 | 0.472 | 1.039 | 0.935 | 1.155 |
| FABP6 | 0.473 | 1.080 | 0.874 | 1.336 |
| CTSD | 0.483 | 1.025 | 0.956 | 1.100 |
| CPB1 | 0.485 | 0.946 | 0.808 | 1.107 |
| ICAM3 | 0.486 | 1.028 | 0.950 | 1.112 |
| CDH5 | 0.486 | 0.973 | 0.901 | 1.051 |
| CD55 | 0.487 | 1.024 | 0.957 | 1.096 |
| GH1 | 0.491 | 1.204 | 0.708 | 2.045 |
| CELA3A | 0.495 | 0.939 | 0.783 | 1.126 |
| CEACAM8 | 0.500 | 1.084 | 0.855 | 1.375 |
| CNPY2 | 0.502 | 1.072 | 0.875 | 1.313 |
| FABP4 | 0.504 | 1.070 | 0.876 | 1.308 |
| EDIL3 | 0.504 | 1.045 | 0.919 | 1.188 |
| MB | 0.504 | 1.051 | 0.907 | 1.218 |
| LTBP2 | 0.510 | 1.035 | 0.933 | 1.149 |
| HSPB1 | 0.511 | 1.093 | 0.837 | 1.426 |
| PROC | 0.512 | 1.030 | 0.942 | 1.127 |
| USP8 | 0.514 | 1.100 | 0.825 | 1.466 |
| CEP43 | 0.516 | 1.090 | 0.840 | 1.414 |
| MFAP5 | 0.524 | 1.043 | 0.916 | 1.186 |
| BPIFB1 | 0.527 | 1.063 | 0.879 | 1.284 |
| CNTN1 | 0.536 | 0.973 | 0.891 | 1.062 |
| GRAP2 | 0.539 | 1.080 | 0.844 | 1.382 |
| PLIN3 | 0.541 | 1.054 | 0.889 | 1.251 |
| CDH6 | 0.548 | 0.961 | 0.842 | 1.096 |
| PLAT | 0.550 | 1.043 | 0.907 | 1.200 |
| AKR1C4 | 0.553 | 1.047 | 0.899 | 1.219 |
| PCSK9 | 0.556 | 1.032 | 0.929 | 1.146 |
| SEMA3F | 0.561 | 0.958 | 0.829 | 1.108 |
| CHL1 | 0.567 | 1.023 | 0.947 | 1.105 |
| CXCL8 | 0.570 | 1.113 | 0.767 | 1.616 |
| GPR37 | 0.577 | 1.067 | 0.848 | 1.344 |
| AGXT | 0.577 | 1.062 | 0.858 | 1.314 |
| SELP | 0.582 | 1.041 | 0.902 | 1.202 |
| GGH | 0.589 | 1.020 | 0.948 | 1.099 |
| GHRL | 0.593 | 1.102 | 0.770 | 1.577 |
| APOM | 0.594 | 1.023 | 0.942 | 1.111 |
| ITGB1 | 0.597 | 0.985 | 0.931 | 1.042 |
| RETN | 0.600 | 1.042 | 0.893 | 1.215 |
| PLA2G1B | 0.606 | 1.036 | 0.904 | 1.187 |
| SERPINE1 | 0.610 | 0.976 | 0.889 | 1.072 |
| CA13 | 0.612 | 1.109 | 0.742 | 1.659 |
| ICAM5 | 0.617 | 1.049 | 0.869 | 1.267 |
| CHRDL2 | 0.619 | 0.939 | 0.733 | 1.204 |
| FAM3C | 0.630 | 0.964 | 0.828 | 1.121 |
| NID1 | 0.642 | 1.024 | 0.926 | 1.133 |
| PLPBP | 0.645 | 1.082 | 0.771 | 1.519 |
| COL4A1 | 0.645 | 0.941 | 0.726 | 1.220 |
| CNST | 0.651 | 0.957 | 0.790 | 1.160 |
| NOTCH1 | 0.662 | 0.989 | 0.941 | 1.039 |
| PCDH17 | 0.663 | 0.971 | 0.850 | 1.110 |
| OLR1 | 0.664 | 1.066 | 0.797 | 1.426 |
| HMOX1 | 0.665 | 0.961 | 0.801 | 1.153 |
| ROR1 | 0.668 | 0.970 | 0.844 | 1.115 |
| SLITRK6 | 0.668 | 1.024 | 0.918 | 1.142 |
| ADGRE5 | 0.669 | 0.981 | 0.896 | 1.073 |
| TCL1B | 0.669 | 1.072 | 0.778 | 1.476 |
| MNDA | 0.671 | 1.097 | 0.713 | 1.689 |
| CD69 | 0.673 | 0.935 | 0.683 | 1.280 |
| VASN | 0.687 | 0.985 | 0.915 | 1.060 |
| IGSF8 | 0.688 | 1.028 | 0.898 | 1.177 |
| ANG | 0.690 | 1.020 | 0.925 | 1.124 |
| THOP1 | 0.691 | 0.961 | 0.791 | 1.169 |
| VCAM1 | 0.691 | 1.015 | 0.942 | 1.093 |
| REN | 0.695 | 1.046 | 0.836 | 1.308 |
| FCGR3B | 0.699 | 0.973 | 0.845 | 1.120 |
| KYAT1 | 0.705 | 1.054 | 0.800 | 1.389 |
| CHIT1 | 0.705 | 0.909 | 0.552 | 1.497 |
| RNASE3 | 0.707 | 1.088 | 0.698 | 1.695 |
| SIGLEC7 | 0.721 | 0.974 | 0.845 | 1.124 |
| LPL | 0.722 | 1.042 | 0.829 | 1.311 |
| S100P | 0.723 | 1.048 | 0.807 | 1.361 |
| ADH4 | 0.729 | 0.958 | 0.753 | 1.220 |
| KIT | 0.738 | 0.985 | 0.901 | 1.077 |
| HYAL1 | 0.739 | 1.011 | 0.946 | 1.081 |
| SPP1 | 0.740 | 1.037 | 0.835 | 1.287 |
| F7 | 0.740 | 1.019 | 0.912 | 1.138 |
| DOK2 | 0.741 | 0.946 | 0.678 | 1.319 |
| CES1 | 0.745 | 0.957 | 0.733 | 1.250 |
| CCDC80 | 0.745 | 1.035 | 0.840 | 1.275 |
| DCTPP1 | 0.750 | 0.975 | 0.836 | 1.138 |
| SEMA7A | 0.751 | 1.013 | 0.934 | 1.099 |
| CST6 | 0.751 | 1.022 | 0.893 | 1.169 |
| CCL16 | 0.758 | 1.023 | 0.883 | 1.186 |
| PCOLCE | 0.759 | 0.979 | 0.851 | 1.125 |
| TP53INP1 | 0.760 | 0.979 | 0.856 | 1.120 |
| PRKAR1A | 0.766 | 0.961 | 0.735 | 1.255 |
| SOD1 | 0.767 | 1.028 | 0.855 | 1.237 |
| GP1BA | 0.771 | 1.014 | 0.922 | 1.116 |
| ITIH3 | 0.775 | 1.027 | 0.856 | 1.231 |
| NRP1 | 0.775 | 0.989 | 0.915 | 1.068 |
| PON2 | 0.778 | 0.985 | 0.886 | 1.095 |
| CPA1 | 0.780 | 0.978 | 0.833 | 1.148 |
| GP2 | 0.781 | 0.969 | 0.775 | 1.212 |
| ITGB2 | 0.783 | 0.988 | 0.903 | 1.080 |
| LILRB5 | 0.785 | 0.975 | 0.813 | 1.169 |
| CBLIF | 0.786 | 0.961 | 0.720 | 1.283 |
| VIM | 0.791 | 1.050 | 0.731 | 1.509 |
| CLTA | 0.792 | 1.027 | 0.841 | 1.255 |
| IGFBPL1 | 0.792 | 1.019 | 0.886 | 1.171 |
| FETUB | 0.796 | 0.987 | 0.892 | 1.092 |
| VSIR | 0.805 | 1.035 | 0.789 | 1.357 |
| MCAM | 0.806 | 0.988 | 0.898 | 1.088 |
| PDCD6 | 0.810 | 1.024 | 0.841 | 1.247 |
| SERPINA12 | 0.813 | 1.014 | 0.903 | 1.139 |
| PTPRS | 0.816 | 1.008 | 0.944 | 1.076 |
| CLEC5A | 0.818 | 1.019 | 0.868 | 1.196 |
| SSC5D | 0.828 | 1.015 | 0.886 | 1.163 |
| SNAP23 | 0.828 | 0.957 | 0.644 | 1.423 |
| ANXA4 | 0.828 | 1.019 | 0.857 | 1.212 |
| IL1RL1 | 0.836 | 0.985 | 0.853 | 1.138 |
| C2 | 0.844 | 1.008 | 0.931 | 1.091 |
| ACY1 | 0.850 | 0.980 | 0.793 | 1.211 |
| CD2AP | 0.855 | 1.031 | 0.741 | 1.436 |
| AHCY | 0.857 | 0.981 | 0.799 | 1.205 |
| PDGFRA | 0.858 | 0.988 | 0.864 | 1.129 |
| CR2 | 0.862 | 1.012 | 0.887 | 1.154 |
| F9 | 0.862 | 1.033 | 0.713 | 1.498 |
| CLEC1A | 0.868 | 1.008 | 0.915 | 1.111 |
| QDPR | 0.869 | 0.983 | 0.802 | 1.205 |
| EPHX2 | 0.877 | 0.984 | 0.796 | 1.215 |
| CA1 | 0.885 | 0.972 | 0.664 | 1.423 |
| DLK1 | 0.890 | 0.988 | 0.835 | 1.169 |
| CD163 | 0.896 | 1.008 | 0.898 | 1.131 |
| NECTIN2 | 0.898 | 0.988 | 0.824 | 1.185 |
| PLA2G2A | 0.900 | 1.010 | 0.867 | 1.176 |
| ST6GAL1 | 0.907 | 1.006 | 0.905 | 1.119 |
| GLO1 | 0.913 | 1.013 | 0.803 | 1.277 |
| SERPINB5 | 0.915 | 1.011 | 0.820 | 1.248 |
| TFRC | 0.917 | 1.005 | 0.920 | 1.097 |
| SDC1 | 0.920 | 0.993 | 0.869 | 1.135 |
| CGREF1 | 0.926 | 0.993 | 0.851 | 1.158 |
| CXCL5 | 0.927 | 1.013 | 0.763 | 1.346 |
| LEPR | 0.932 | 0.996 | 0.901 | 1.100 |
| MEGF9 | 0.933 | 0.998 | 0.942 | 1.056 |
| PLXNB3 | 0.939 | 0.994 | 0.842 | 1.173 |
| HEBP1 | 0.946 | 1.007 | 0.819 | 1.239 |
| CRHR1 | 0.946 | 0.992 | 0.785 | 1.254 |
| SPON2 | 0.947 | 0.994 | 0.823 | 1.200 |
| PEAR1 | 0.947 | 1.002 | 0.939 | 1.070 |
| AK1 | 0.949 | 1.012 | 0.695 | 1.475 |
| FCN2 | 0.952 | 1.004 | 0.878 | 1.148 |
| ANGPTL1 | 0.957 | 0.997 | 0.892 | 1.114 |
| CTF1 | 0.969 | 0.995 | 0.760 | 1.303 |
| BAG6 | 0.975 | 1.002 | 0.865 | 1.162 |
| ACE2 | 0.978 | 1.003 | 0.816 | 1.233 |
| DPT | 0.978 | 0.999 | 0.910 | 1.096 |
| IL19 | 0.981 | 1.002 | 0.817 | 1.231 |
| CLC | 0.982 | 1.003 | 0.781 | 1.287 |
| SFTPD | 0.984 | 0.998 | 0.782 | 1.272 |
| FCRL1 | 0.986 | 0.998 | 0.827 | 1.205 |
| ANPEP | 0.992 | 1.000 | 0.931 | 1.075 |
| ENPP2 | 0.993 | 1.000 | 0.902 | 1.109 |
| PLTP | 0.997 | 1.000 | 0.897 | 1.115 |

1. Collins, G.S., et al., *Transparent reporting of a multivariable prediction model for individual prognosis or diagnosis (TRIPOD): the TRIPOD Statement.* BMC Medicine, 2015. **13**(1): p. 1.

2. MacDonald, T.M., et al., *Randomized trial of switching from prescribed non-selective non-steroidal anti-inflammatory drugs to prescribed celecoxib: the Standard care vs. Celecoxib Outcome Trial (SCOT).* Eur Heart J, 2017. **38**(23): p. 1843-1850.

3. Kragelund, C. and T. Omland, *A farewell to body-mass index?* The Lancet, 2005. **366**(9497): p. 1589-1591.

4. Murray, S., *Is waist-to-hip ratio a better marker of cardiovascular risk than body mass index?* CMAJ : Canadian Medical Association journal = journal de l'Association medicale canadienne, 2006. **174**(3): p. 308-308.

5. Franklin, S.S. and N.D. Wong, *Hypertension and Cardiovascular Disease: Contributions of the Framingham Heart Study.* Global Heart, 2013. **8**(1): p. 49-57.

6. Vittinghoff, E. and C.E. McCulloch, *Relaxing the Rule of Ten Events per Variable in Logistic and Cox Regression.* American Journal of Epidemiology, 2007. **165**(6): p. 710-718.

7. Assarsson, E., et al., *Homogenous 96-plex PEA immunoassay exhibiting high sensitivity, specificity, and excellent scalability.* PLoS One, 2014. **9**(4): p. e95192.

8. Ahmetaj-Shala, B., et al., *Development of a novel UHPLC-MS/MS-based platform to quantify amines, amino acids and methylarginines for applications in human disease phenotyping.* Sci Rep, 2018. **8**(1): p. 13987.

9. van den Berg, V.J., et al., *IgM anti-malondialdehyde low density lipoprotein antibody levels indicate coronary heart disease and necrotic core characteristics in the Nordic Diltiazem (NORDIL) study and the Integrated Imaging and Biomarker Study 3 (IBIS-3).* EBioMedicine, 2018. **36**: p. 63-72.

10. Khan, T.Z., et al., *Oxidised LDL and Anti-Oxidised LDL Antibodies Are Reduced by Lipoprotein Apheresis in a Randomised Controlled Trial on Patients with Refractory Angina and Elevated Lipoprotein(a).* Antioxidants (Basel), 2021. **10**(1).
